# A workflow of massive identification and application of intron markers using snakes as a model

**DOI:** 10.1002/ece3.3525

**Published:** 2017-10-22

**Authors:** Jiang‐Ni Li, Chong He, Peng Guo, Peng Zhang, Dan Liang

**Affiliations:** ^1^ State Key Laboratory of Biocontrol College of Ecology and Evolution School of Life Sciences Sun Yat‐sen University Guangzhou China; ^2^ College of Life Sciences and Food Engineering Yibin University Yibin China

**Keywords:** *Gloydius*, intron, noncoding, phylogeny, Serpentes

## Abstract

Relative to the commonly used mitochondrial and nuclear protein‐coding genes, the noncoding intron sequences are a promising source of informative markers that have the potential to resolve difficult phylogenetic nodes such as rapid radiations and recent divergences. Yet many issues exist in the use of intron markers, which prevent their extensive application as conventional markers. We used the diverse group of snakes as an example to try paving the way for massive identification and application of intron markers. We performed a series of bioinformatics screenings which identified appropriate introns between single‐copy and conserved exons from two snake genomes, adding particular constraints on sequence length variability and sequence variability. A total of 1,273 candidate intron loci were retrieved. Primers for nested polymerase chain reaction (PCR) were designed for over a hundred candidates and tested in 16 snake representatives. 96 intron markers were developed that could be amplified across a broad range of snake taxa with high PCR successful rates. The markers were then applied to 49 snake samples. The large number of amplicons was subjected to next‐generation sequencing (NGS). An analytic strategy was developed to accurately recover the amplicon sequences, and approximately, 76% of the marker sequences were recovered. The average *p*‐distances of the intron markers at interfamily, intergenus, interspecies, and intraspecies levels were .168, .052, .015, and .004, respectively, suggesting that they were useful to study snake relationships of different evolutionary depths. A snake phylogeny was constructed with the intron markers, which produced concordant results with robust support at both interfamily and intragenus levels. The intron markers provide a convenient way to explore the signals in the noncoding regions to address the controversies on the snake tree. Our improved strategy of genome screening is effective and can be applied to other animal groups. NGS coupled with appropriate sequence processing can greatly facilitate the extensive application of molecular markers.

## 1 INTRODUCTION

Molecular phylogenetic studies rely mostly on analyzing the signals in DNA sequences to gain information on organisms’ evolutionary relationships (Delsuc, Brinkmann, & Philippe, 2005; Thomson, Wang, & Johnson, 2010). The widely and frequently used mitochondrial genes/genome have the drawbacks of close linkage and maternal inheritance. Different mitochondrial markers even the whole mitochondrial genome are only viewed as one locus in phylogenetic analyses. The maternal signals they carry often cannot reflect the true species evolutionary histories. Alternatively, markers can be developed from the nuclear genome. Analyzing multiple independent nuclear markers that are inherited biparentally will alleviate stochastic effects and is generally thought to provide more reliable estimates for the organisms’ evolutionary history. Nuclear protein‐coding (NPC) regions have been extensively explored because they are relatively conserved which makes assessing orthology and aligning across divergent species straightforward. Widely applicable primers can be designed for NPC markers (Che et al., 2017; Li, Ortí, Zhang, & Lu, 2007; Shen, Liang, Feng, Chen, & Zhang, 2013; Wahlberg & Wheat, 2008). However, the conservativeness of NPC regions also means that they may not have sufficient phylogenetic signals to resolve rapid divergences or shallow relationships. A recent study of the phylogeny of Laurasiatherian mammals has shown that even a genome‐scale data set of coding sequences could not satisfactorily produce robust and congruent results for this ancient rapid radiation event (Chen, Liang, & Zhang, 2017).

Compared with the constrained coding regions, noncoding regions evolve faster and potentially carry a larger number of phylogenetic signals desirable for treating rapid radiations and recent divergence events (Dalebout, Steel, & Baker, 2008; Foley et al., 2015; Pons, Barraclough, Theodorides, Cardoso, & Vogler, 2004; Schröder, Bleidorn, Hartmann, & Tiedemann, 2009; Wang et al., 2012; Yu et al., 2011). Moreover, while coding regions have more or less been affected by the action of selection, noncoding regions can generally be considered evolving neutrally, which is more consistent with the evolutionary assumptions underlying phylogenetic tree construction. Inferences from noncoding regions are thus less affected by convergent evolution. There are several ways to explore the signals in noncoding sequences, for instances, designing anonymous nuclear markers (ANM) from random draws of the genome, obtaining flanking sequences (mostly noncoding) of the ultraconserved elements (UCEs) through capture, and investigating introns, the noncoding regions in genes. In practical terms, introns have many advantages. The region of an intron is defined accurately by the flanking exons but not by length which, like that of other noncoding sequences, may vary greatly in different species. The conserved flanking exons can also help in determining the orthology of introns, which is difficult for other noncoding sequences. It is convenient to find priming sites in the flanking exons and amplify across the target intron. The so‐called exon‐primed intron‐crossing (EPIC) markers can usually be applied within a specific clade or broader. Besides phylogenetic analysis, the informative intron markers are also useful in population genetics and species delimitation (Dool et al., 2016; Egea et al., 2016; Yu et al., 2011). From the ever‐growing genome data, intron markers have been developed for several animal groups (Backström, Fagerberg, & Ellegren, 2008; Chenuil et al., 2010; Igea, Juste, & Castresana, 2010; Li, Riethoven, & Ma, 2010; Rodríguez‐Prieto, Igea, & Castresana, 2014), but are still limited for many other clades with unsolved relationships. Various issues exist in the application of intron markers regarding PCR optimization, sequencing, sequence alignment, etc., which confined the number of intron markers used in most studies and prevent their extensive application as conventional markers.

Here, we used snakes as an example in an attempt to pave the way for massive identification and application of intron markers. Snakes are a diverse group with approximately 3,500 currently recognized species found on every continent (except Antarctica) and many islands (Streicher & Wiens, 2016; Wiens et al., 2012; Zheng & Wiens, 2015). The advanced snakes, which include all known dangerously venomous species, are one of the largest radiations of terrestrial vertebrates (Pyron et al., 2011, 2014). A reliable snake phylogenetic framework has not yet been achieved, although many efforts have been made in the past two decades using several mitochondrial gene fragments, multiple nuclear exons, and numerous loci from UCE capture (Alencar et al., 2016; Figueroa, Mckelvy, Grismer, Bell, & Lailvaux, 2016; Lawson, Slowinski, Crother, & Burbrink, 2005; Lee, Lee, Sanders, & Palci, 2016; Pyron, Burbrink, & Wiens, 2013; Pyron et al., 2011, 2014; Streicher & Wiens, 2016; Wiens et al., 2012; Zheng & Wiens, 2015). While the resolution and support have progressively been improved, there are still controversies in many parts of the snake tree, such as the subfamilial relationships within Lamprophiidae and Colubridae and other shallower‐scale relationships (Figueroa et al., 2016; Pyron et al., 2013; Wiens et al., 2012). On the other hand, introns have been tried in several studies, which appear to effectively increase the resolution of the inferred phylogenetic tree. For example, an analysis with two introns provided support for the inclusion of the subclade consisting of *Trimeresurus macrops* and *T. venustus* within the “albolabris” group (Creer, Pook, Malhotra, Thorpe, & Lee, 2006). Malhotra, Creer, Pook, and Thorpe (2010) reported that adding three nuclear intron sequence data helped to identify the Asian sister group of New World pit vipers. Despite their fairly good performance, until now the intron markers available for snakes have been rare, hindering their wider application to snake phylogenetics.

To explore the phylogenetic signals in intron sequences, we screened the genome sequences of two snakes, that is, the Burmese python (*Python molurus bivittatus*) and the king cobra (*Ophiophagus hannah*) (Castoe et al., 2013; Vonk et al., 2013), with a bioinformatic pipeline adapted from previous studies, and targeted a suite of introns encompassed by single‐copy and conserved exons. We tested over a hundred of the potential intron loci in 16 snake representatives across a broad taxonomic range and developed 96 universal intron markers for snakes. The markers were then applied to 49 snake samples with nested PCR that yielded high successful rates with no need for further optimization. We sequenced the large number of amplicons using NGS to reduce labor intensity. An analytic strategy was developed to accurately recover the amplicon sequences. Our results demonstrate that the strategies we applied on genome screening and sequence processing are effective, and the newly developed intron markers are informative and useful for snake phylogenetic studies. The coupling with NGS can greatly facilitate the extensive application of molecular markers.

## 2 MATERIALS AND METHODS

### 2.1 Taxon sampling and DNA extraction

A total of 49 snake specimens were sampled, encompassing 31 species, 19 genera, and 10 families (Table 1). To develop universal markers for snakes, 16 specimens that spanned a wide taxonomic range were first chosen to test the experimental performance of the designed primers (see Table 1 for details). The successful markers were then applied to all 49 specimens to construct a snake phylogeny. Among the 49 specimens, 29 were of the genus *Gloydius* (Serpentes: Crotalinae) representing 11 species, for demonstrating the utility of these markers in addressing shallow‐scale phylogenetics.

**Table 1:** List of all species used in this study

Family	Genus	Species	Collection locality or source	Voucher
Typhlopidae	*Indotyphlops*	*Indotyphlops braminus* a	Hongkong, China	RE28
Boidae	*Eryx*	*Eryx tataricus* a	Private breeding	RE37
Pythonidae	*Python*	*Python regius* a	—	RE26
Xenopeltidae	*Xenopeltis*	*Xenopeltis unicolor* a	Mengla, Yunnan, China	RE22
Xenoderrmatidae	*Achalinus*	*Achalinus rufescens* a	Private breeding	RE51
Pareatidae	*Pareas*	*Pareas margaritophorus* a	Bawanglin, Hainan, China	RE47
Homalopsidae	*Myrrophis*	*Myrrophis chinensis* a	Shaoguan, Guangdong, China	RE12
Elapidae	*Naja*	*Naja atra* a	Shaoguan, Guangdong, China	RE04‐2
Elapidae	*Bungarus*	*Bungarus multicinctus* a	Mengla, Yunnan, China	RE17
Colubridae	*Xenochrophis*	*Xenochrophis piscator* a	Shaoguan. Guangdong, China	RE35
Colubridae	*Oligodon*	*Oligodon lacroixi* a	—	RE41
Colubridae	*Amphiesma*	*Amphiesma boulengeri*	Shaoguan, Guangdong, China	RE55
Colubridae	*Elaphe*	*Elaphe carinata* a	Shaoguan, Guangdong, China	RE59
Viperidae	*Daboia*	*Daboia siamensis* a	Guangzhou, Guangdong, China	RE49
Viperidae	*Azemiops*	*Azemiops feae*	Guangzhou, Guangdong, China	RE60
Viperidae	*Deinagkistrodon*	*Deinagkistrodon aeutus*	Shaoguan, Guangdong, China	RE05
Viperidae	*Viridovipera*	*Viridovipera stejnegeri*	Yongzhou, Hunan, China	RE46
Viperidae	*Viridovipera*	*Viridovipera gumprechti* a	Mengla, Yunnan, China	RE21
Viperidae	*Protobothrops*	*Protobothrops jerdonii*	—	RE44
Viperidae	*Protobothrops*	*Protobothrops mucrosquamatus* a	Shaoguan, Guangdong, China	RE56
Viperidae	*Gloydius*	*Gloydius brevicaudus* a	—	RE45
Viperidae	*Gloydius*	*Gloydius brevicaudus*	Huangshan, Anhui, China	GP01
Viperidae	*Gloydius*	*Gloydius brevicaudus*	Kuangdian. Liaoning, China	GP02
Viperidae	*Gloydius*	*Gloydius brevicaudus*	Hebei, China	GP03
Viperidae	*Gloydlus*	*Gloydius brevicaudus*	Hunan, China	GP05
Viperidae	*Gloydius*	*Gloydius brevicaudus*	Huangshan, Anhui, China	GP06
Viperidae	*Gloydius*	*Gloydius brevicaudus*	Anhui, China	GP07
Viperidae	*Gloydius*	*Gloydius ussuriensis*	Liaoning, China	GP08
Viperidae	*Gloydius*	*Gloydius ussuriensis*	Ji'an, Jilin, China	GP09
Viperidae	*Gloydius*	*Gloydius ussuriensis*	—	GP10
Viperidae	*Gloydius*	*Gloydius ussuriensis*	Tonghua, Jilin, China	GP11
Viperidae	*Gloydius*	*Gloydius ussuriensis*	Tonghua, Jilin, China	GP12
Viperidae	*Gloydius*	*Gloydius intermedins*	Yan'an, Shaanxi, China	GP15
Viperidae	*Gloydins*	*Gloydius intermedins*	Yan'an, Shaanxi, China	GP16
Viperidae	*Gloydius*	*Gloydius saxalilis*	Benxi, Liaoning, China	GPI7
Viperidae	*Gloydius*	*Gloydius lijianlii*	Shandong, China	GP18
Viperidae	*Gloydius*	*Gloydius lijianlii*	Yantai, Shandong, China	GP19
Viperidae	*Gloydius*	*Gloydius saxalilis*	Ji'an, Jilin, China	GP20
Viperidae	*Gloydius*	*Gloydius saxatilis*	Jilin, China	GP2I
Viperidae	*Gloydius*	*Gloydius shedaoensis*	Shedao island, Dalian, China	GP22
Viperidae	*Gloydius*	*Gloydius shedaoensis*	Shedao island, Dalian, China	GP23
Viperidae	*Gloydius*	*Gloydius liupanensis*	Liupanshan, Ningxia, China	GP24
Viperidae	*Gloydius*	*Gloydius strauchi*	Shiqu, Sichuan, China	GP25
Viperidae	*Gloydius*	*Gloydius qinglingensis*	Zhouzhi, Shaanxi, China	GP27
Viperidae	*Gloydius*	*Gloydius qinglingensis*	Shaanxi, China	GP28
Viperidae	*Gloydius*	*Gloydius blomhoffi*	Teuriisland, Hokkaido, Japan	GP29
Viperidae	*Gloydius*	*Gloydius monticola*	Shangri‐La, Yunnan, China	GP30
Viperidae	*Gloydius*	*Gloydius monticola*	Shangri‐La, Yunnan, China	GP31
Viperidae	*Gloydius*	*Gloydius strauchi*	Shiqu, Sichuan, China	GP33

a Species were chosen to verify the effectiveness of the newly‐designed primers.

Total genomic DNA was extracted from tissue samples (muscle, scale, or blood) with a TIANamp Genomic DNA Kit (TIANGEN Inc., Beijing, China) following the manufacturer's instructions. All the genomic DNA was diluted to a concentration of 30 ng/μl with water and stored at −20°C before PCR amplification.

### 2.2 Bioinformatic mining of potential intron markers for snakes

The genome sequences of the king cobra (*Ophiophagus hannah*) and the Burmese python (*Python molurus bivittatus*) were downloaded from the NCBI database in FASTA format. The exon sequences and the genome sequences of the anole lizard (*Anolis carolinensis*) were downloaded from the Ensembl database.

Our strategy for developing intron markers was similar to that in Li et al. (2010) with additional blast steps to ensure “single‐copy” exons and more constraints on intron lengths and sequence variability to identify marker candidates with sufficient signals and easy to manipulate in routine PCR. First, exons containing the 5′‐ or 3′‐ untranslated regions (UTRs) were removed to avoid high variability in subsequent alignments. The remaining exons were blasted against themselves, and only those having but one hit above the threshold of 30% coverage and 60% similarity were retained. The single‐copy exons from the anole lizard were then blasted against the king cobra and the Burmese python genome sequences, respectively, with an e‐value threshold of 10^−5^ to identify orthologous exons (coverage larger than 70% and identity larger than 80%) in snakes. Then, the resulting orthologous exons in the two snakes were blasted against their corresponding genomes to insure “single‐copy” in snakes. To design nested PCR primers, exons smaller than 100 bp in at least one snake were discarded. Next, according to the location of the single‐copy conserved exons, we screened for exon‐exon pairs separated by introns with sizes ranging from 500 to 1,500 bp for easy management in routine PCR amplification. Besides the above steps, we considered that homologous intron lengths could vary greatly in different species, thus calculated the relative standard deviation (RSD) of the length of the corresponding introns in the two snake species and retained those of which length RSD was smaller than 10%. The FASTA sequences of the introns were retrieved from each genome. We also screened and discarded introns of which the sequences were very similar in the two snakes (identity >0.9) to ensure sufficient signals. If multiple candidates were present within the range of 1,500 bp, the whole region was treated as one marker candidate.

### 2.3 Primer design and validation

A total of 130 marker candidates were chosen randomly for primer design and validation. The exon portion of the sequences was aligned with ClustalW (Higgins, Thompson, & Gibson, 1996) based on the translated amino acid sequence. In the conserved exon regions of each candidate, we designed two pairs of degenerate primers to apply nested PCR which has been shown to be more effective than conventional PCR in obtaining target sequences (Shen, Liang, & Zhang, 2012; Shen et al., 2013). Whenever possible, the 3′ end of the primers was designed at the 1st and 2nd codon position, and the degeneracy of the second‐round primers was minimized to increase reaction specificity.

To verify the effectiveness of the primers we had designed, they were assayed in a set of “test taxa” including 16 snake representatives (see Table 1 for details). Conditions of the nested PCR are similar to that in Shen et al. (2013) except that the cycle number of the second‐round PCR was set to 30. The PCR products were then subjected to agarose gel electrophoresis. If more than 10 of the 16 test taxa gave rise to target amplicon bands, the primer pairs were considered qualified.

### 2.4 High‐throughput sequencing of the amplicons from 49 snake samples and data processing

The newly developed markers were applied to all 49 snake samples with nested PCR performed as stated above.

For HiSeq sequencing, the PCR products were processed following the procedure described in Feng, Liu, Chen, Liang, and Zhang (2016) with some modifications. Briefly, the amplicons of different markers for a species were pooled, and the pooled PCR products were randomly fragmented and tagged with barcode linkers at both ends. The fragmented, barcode‐added PCR product mixtures of different species were size selected (200–500 bp) with Mag‐Beads (Beckman Coulter, Inc.). Then, the target fragments were enriched with a 10‐cycle PCR. The PCR products of different species were pooled into one library according to the quantification on agarose gel electrophoresis and sequenced on an Illumina HiSeq2000 platform after final purification with Mag‐Beads and gel cutting.

After sequencing, the paired‐end reads were sorted according to the species‐specific barcode linkers. The sorted reads of each species were assembled using the de novo transcriptome assembler TRINITY (Grabherr et al., 2011) under the “Pasafly” algorithm that is recommended to lower assembly chimeras. To ensure high sequence quality, only contigs with an average sequencing depth ≥10 (calculated using BOWTIE (Langmead, Trapnell, Pop, & Salzberg, 2009) and SAMTOOLS (Li et al., 2009)) were retained for subsequent analysis. The 96 marker sequences retrieved from the king cobra's genome were used as references to call the orthologous markers in each of the sequenced species by performing BLASTN against the contig sequences (*e*‐value <1e‐5, identity >60%). If a reference sequence had multiple hits in one species, we usually retained the best hit with the highest score but occasionally the second best hit when its average sequencing depth was more than three times over the best hit. Then, a reversed BLASTN was performed with the contigs obtained from the aforementioned steps against the reference sequences to identify potential chimeras (*e*‐value <1e‐5, identity >60%). After these steps, all locus bins contained no more than one contig for one species. The orthologous contigs of one marker from different species as well as the reference sequence from the king cobra were aligned using the SATé iterative alignment program (Liu et al., 2012) with MAFFT (Katoh, Kuma, Toh, & Miyata, 2005; Katoh & Toh, 2008) as the aligner, OPAL (Wheeler & Kececioglu, 2007) as the merger, FastTree (Price, Dehal, & Arkin, 2010) as the tree estimator, and other parameters under the default SATé‐II‐fast settings. The sequences assembled exceeding the priming sites at both ends of each contig were trimmed according to the reference sequence. To refrain potential chimeras, we further controlled the orthologous sequence length to be within 0.3–2 times the respective reference sequence length. Furthermore, we calculated the mean *p‐*distance of each contig with the other contigs of one marker and made comparisons. Contigs with deviating mean *p‐*distance values were more variable than the other orthologs, which were likely rogue sequences and were therefore removed. Finally, single‐gene trees were built using RaxML v8.0.0 (Stamatakis, 2014) under the model of GTR + Γ with anolis as outgroup and checked manually. Sequences that had long branches and/or occurred more basal than the outgroup were discarded. Note that the sequences we obtained were consensus sequences of each sample for each marker.

### 2.5 Calculation of the relative evolutionary rate of the newly developed markers

The average *p‐*distances of each marker were calculated at four different taxonomic levels: interfamily, intergenus, interspecies, and intraspecies. For the interfamily level, we compared the orthologous markers from any two species that were not in the same family; for the intergenus level, markers from species that were in different genera of the same family were compared; for the interspecies level, markers from any two species of the same genus were compared; and for the intraspecies level, we compared the orthologous markers from individuals of the same species.

### 2.6 Topological comparisons

The pairwise distances of the tree topologies generated from the intron markers and the concatenated topology were calculated based on Robinson–Foulds metric (RF‐distance) using Pankey's (2014) Python script posted at https://scriptomika.wordpress.com/2014/01/27/59/. The tree‐to‐tree distances were then visualized using multidimensional scaling (MDS) in R (Hillis, Heath, & St, 2005).

### 2.7 Phylogenetic analyses

Alignments of all intronic marker sequences from the snake samples were made using the SATé iterative alignment program with parameter settings as stated above. The alignments were further refined using Gblocks v0.91b (Castresana, 2000) (allowed gap positions = all). All refined alignments were combined into a concatenated data set. Maximum‐likelihood (ML) analysis was performed using RAxML v8.0.0 (Stamatakis, 2014) with the GTR + Γ nucleotide model under 96 partitions. Branch support for the resulting phylogeny was evaluated with 500 bootstrapping replicates. The multiple‐species coalescent‐based (MSC) analysis was performed using ASTRAL 4.7.12 (Mirarab et al., 2014), which was supplied with the gene trees estimated for each marker by RAxML with the model of GTR + GAMMA.

## 3 RESULTS

### 3.1 96 novel intron markers were developed from the snake genomes

**Figure 1:**
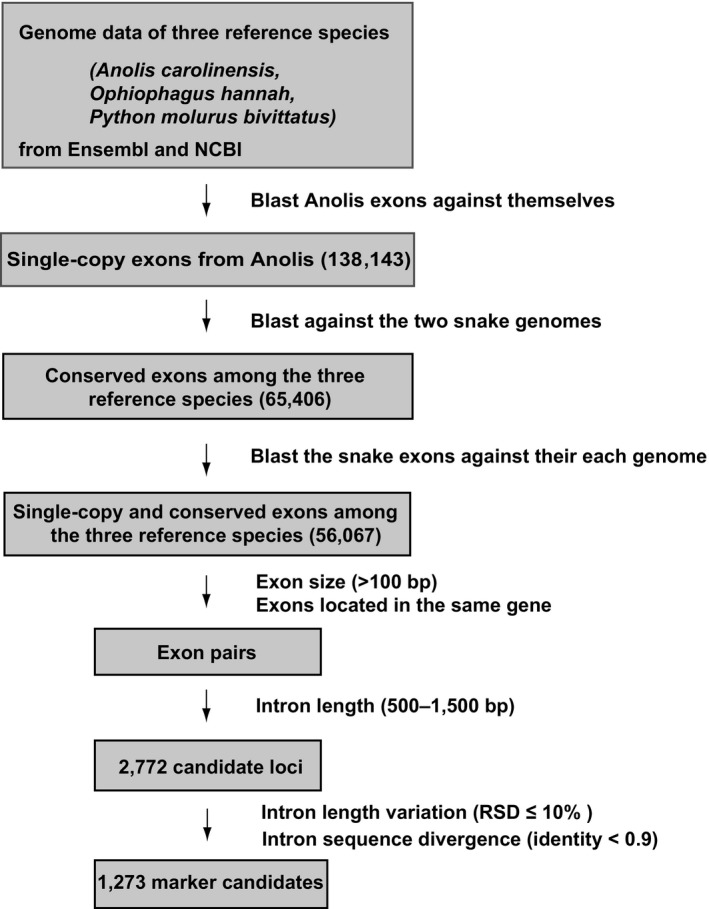
Scheme of the bioinformatic pipeline

To screen for potential intron regions as markers for snake phylogeny, we analyzed the genome sequences of two snake representatives (the Burmese python and the king cobra) and their close relative, the anole lizard. Following the EPIC strategy, primers were to design in the conserved regions within the exons that flanked the target introns. Therefore, a key step was to screen for single‐copy or low‐copy exons conserved in the three reference species, which could greatly lower the risk of nontarget amplification of paralogous loci. Next, length selections (>100 bp in both snakes) were applied to the exons to ensure enough regions for nested PCR primer design. The qualified exons, according to their locations, if close to each other in the same gene, were recorded as a pair. Then, the intron length within an exon pair was further confined to 500–1,500 bp in at least one snake for easy amplification by routine PCR. At this step, a total of 2,772 exon pairs were obtained. Because the lengths of the orthologous introns in different species were often not stable, we discarded the sequences of which the intron lengths varied greatly in the two snakes, hoping to alleviate marker length variation among different snake species and facilitate subsequent sequence alignment. We also removed the sequences of which the intron parts were very similar in the two snakes to ensure sufficient phylogenetic information of the markers. Finally, 1,273 potential intron markers were retrieved (Figure 1).

We randomly chose 130 marker candidates and designed nested PCR primers for them. The primers were then tested in 16 snake species that spanned a wide taxonomic range for their universal utility in PCR. After agarose gel electrophoresis, 96 of the primers were considered appropriate according to our criterion that more than 10 of the 16 test taxa gave rise to target amplicon bands. These markers were each located in different protein‐coding genes. They were named by combining the name of the gene they were located in and the sequence numbers of the exons flanking them. The lengths of these 96 intron markers ranged from 510 to 1,608 bp, with an average length of 917 bp (referring to the sequences in the king cobra, see Table 2 for details). They all had a low GC content, as typically observed in noncoding sequences (Table 2). The primer sequences for each of the markers are listed in Table S2.

**Table 2:** Information for the 96 novel intron markers

Loci	Length (bp)	%GC mean	Mean *p*‐distance	Taxa amplified (%)	Loci	Length (bp)	%GC mean	Mean *p*‐distance	Taxa amplified (%)
ARHGAP4_8‐9	926	38.4	.070	49 (100)	AGBL5_6‐7	627	40.0	.057	46 (93)
ASXL3_9‐10	1,035	32.1	.053	49 (100)	C6orf62_4‐5	690	35.3	.083	46 (93)
CACHD1_12‐13	1,146	35.2	.071	49 (100)	CPOX_1‐2	568	35.2	.084	46 (93)
CASC3_5‐6	613	36.7	.067	49 (100)	CSTF1_2‐3	803	32.3	.060	46 (93)
CELSR3_21‐23	1,166	44.4	.061	49 (100)	GOT1_4‐5	648	37.8	.082	46 (93)
DARS_8‐9	1,206	39.9	.060	49 (100)	LZTR1_14‐15	775	34.9	.064	46 (93)
DICER1_3‐4	629	35.7	.063	49 (100)	NUTM1_1‐2	1,580	41.9	.062	46 (93)
E1F4G2_14‐15	626	36.1	.057	49 (100)	SLU7_14‐15	769	40.3	.070	46 (93)
HUWE1_56‐57	760	40.1	.066	49 (100)	U2AF2_11‐12	911	39.5	.103	46(93)
INO80_9‐11	766	32.0	.073	49 (100)	UGCG_4‐5	1,235	36.9	.061	46 (93)
IPO9_14‐15	632	33.1	.066	49 (100)	VPS13B_36‐37	885	36.7	.101	46 (93)
NIPBL_6‐7	802	34.5	.067	49 (100)	ZNF608_3‐4	1,388	37.2	.068	46 (93)
RALGAPB_6‐7	987	31.6	.069	49 (100)	ANAPC1_2‐3	1,154	31.8	.068	45 (91)
SAP18_2‐3	1,116	33.5	.057	49 (100)	C6orf52_3‐4	1,140	31.0	.071	45 (91)
SRP72_2‐4	915	34.1	.084	49 (100)	LYST_45‐46	796	36.9	.079	45 (91)
SULF1_7‐8	646	36.0	.060	49 (100)	MYOF_36‐37	958	36.3	.072	45 (91)
TBC1D16_9‐10	675	44.3	.079	49 (100)	PDXK_3‐4	1,339	35.1	.057	45 (91)
UBXN7_4‐6	763	32.0	.065	49 (100)	POLR1B_4‐5	1,451	37.6	.054	45 (91)
AQR_17‐18	963	35.3	.096	48 (97)	RBM22_5‐6	881	33.5	.078	45 (91)
CLINT1_2‐3	1,136	37.9	.070	48 (97)	SEC13_6‐7	961	35.7	.063	45 (91)
CNOT10_11‐12	877	32.3	.074	48 (97)	SMC2_22‐23	773	37.0	.082	45(91)
DDB1_16‐I8	781	36.4	.076	48 (97)	YIPF3_2‐3	710	35.7	.072	45 (91)
DDX23_9‐10	553	38.1	.082	48 (97)	HIPK1_11‐12	836	37.5	.078	44 (89)
DLST_1‐2	769	35.5	.072	48 (97)	SF3B1_18‐19	624	34.3	.180	44 (89)
DNMT1_16‐17	1,034	42.7	.091	48 (97)	WDFY3_62‐63	1,284	41.1	.077	44 (89)
DYNC1H1_69‐70	857	33.7	.066	48 (97)	WDR36_7‐8	651	35.7	.071	44 (89)
JMJD6_2‐3	1,563	36.1	.059	48 (97)	PSMD12_3‐4	511	30.1	.050	43 (87)
PRR7_1‐2	871	50.9	.051	48 (97)	UBE3A_4‐5	1,328	34.0	.065	43 (87)
RIOK3_8‐9	943	32.9	.092	48 (97)	USP34_75‐77	1,258	33.1	.075	43 (87)
RUVBL2_7‐9	939	43.8	.096	48 (97)	CNOT2_13‐14	703	30.7	.061	42 (85)
SKIV2L2_22‐23	978	30.0	.014	48 (97)	MFSD4_6‐7	770	35.2	.080	42 (85)
SSRP1_5‐6	542	35.8	.084	48 (97)	MCM2_3‐4	1,224	43.2	.069	41 (83)
TMCO3_2‐3	870	30.7	.074	48 (97)	TENM3_5‐6	687	29.8	.077	41 (83)
TOMM70A_4‐5	1 133	35.9	.073	48 (97)	PRPF38A_3‐5	601	30.3	.110	40 (81)
TRAPPC11_28‐29	685	33.7	.069	48 (97)	POLR2B_4‐5	686	30.4	.098	39 (79)
WDR47_13‐14	691	34.4	.075	48 (97)	TAF2_10‐11	951	34.7	.087	39 (79)
ZNF142_4‐5	1,280	44.8	.057	48 (97)	COPB2_14‐15	617	34.3	.093	37 (75)
CXXC1_1‐2	1,292	33.9	.084	47 (95)	KIF3A_12‐13	1,454	38.3	.120	36 (73)
DCAF7_2‐3	811	34.4	.061	47 (95)	KLHL24_2‐3	800	30.9	.054	36 (73)
DDX18_7‐8	598	32.9	.086	47 (95)	MYOID_7‐8	1,003	30.4	.145	32 (65)
DYNC2H1_43‐44	1,608	36.3	.070	47 (95)	ACTR8_1‐2	644	37.3	.095	30 (61)
FTSJ3_2‐3	631	42.7	.077	47 (95)	PTPRQ_4‐5	883	32.0	.095	21 (42)
MPDZ_4‐5	1,288	39.4	.094	47 (95)	PXK_9‐10	684	34.4	.125	21 (42)
UBA1_19‐22	935	41.3	.062	47 (95)	B1RC6_17‐18	1,044	34.1	.143	18 (36)
UBR5_13‐14	758	31.3	.063	47 (95)	HBS1L_7‐8	1,136	32.3	.112	18 (36)
USP14_2‐4	1,036	31.8	.070	47 (95)	ERP44_5‐6	954	31.2	.185	17 (34)
VPS54_6‐7	689	33.4	.071	47 (95)	TTN_89‐90	1,359	32.6	.161	16 (32)
ZSWIM8_26‐27	569	39.4	.135	47 (95)	FOXK2_5‐6	1,244	31.7	.021	12 (24)

Markers are ranked by the successful rate of amplification among all 49 samples.

### 3.2 Performance and characteristics of the newly developed intron markers

To demonstrate the phylogenetic performance of the 96 newly developed intron markers, we amplified and sequenced them in 49 snake samples (Table 1) to construct a snake phylogeny. We intentionally included 29 specimens of the genus *Gloydius* as an example to assess the performance of these markers at shallow evolutionary depth. Agarose gel electrophoresis showed that the PCR successful rates were generally high (average 89.6%) with 82 markers over 80% (Table 2). Considering our broad sample range (from Typhlopidae to Colubridae), this result reflected the universal usefulness of most of our newly developed primers for snakes. Additionally, approximately 98% of the PCR reactions produced a single band, indicating that our single‐copy exon screening step was effective and that heterozygotic length variation was infrequent. Assembly of the HiSeq sequencing data yielded 3,940 (~84%) of the 4,704 sequences (96 loci × 49 samples). After further screening with stringent criteria to remove potential chimeras, 3,591 (~76%) sequences were finally considered valid. From the angle of the specimens, 44 of them had a sequence recovery rate between 60% and 90%. The low sequence recovery rates (~49%) of the remaining five specimens were most likely due to the poor quality of the DNA extracted from them.

**Figure 2:**
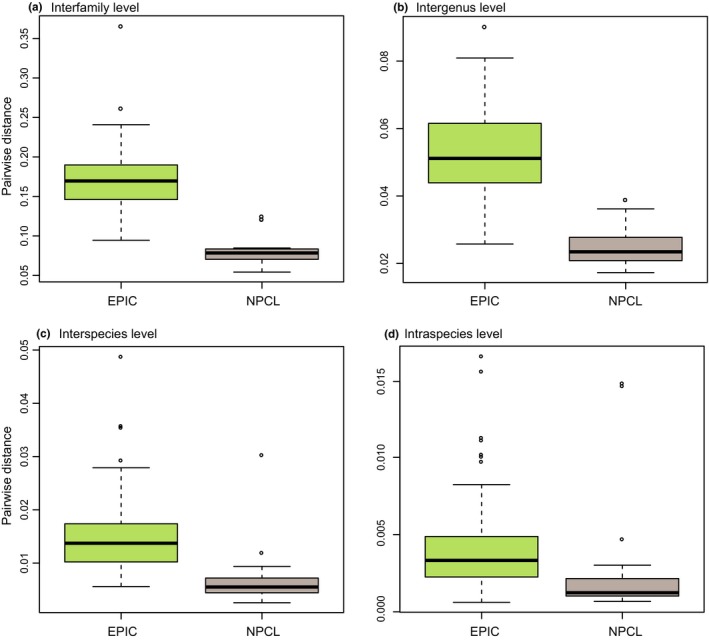
Comparison of the mean pairwise distance between our intron markers and the Nuclear protein‐coding markers among different snake taxa at four different taxonomic levels (a. interfamily, b. intergenus, c. interspecies, and d. intraspecies)

The evolutionary rate, as evidenced by the degree of variability, is an important parameter of a marker that determines its applicability for different phylogenetic questions. The mean *p‐*distance for each of the 96 markers among all 49 samples was calculated and listed in Table 2 of which 83 were over 0.06. We also amplified and sequenced 19 universal NPC markers (Shen et al., 2013) in the snake samples (our unpublished data). When plotted and compared at four taxonomic levels (interfamily, intergenus, interspecies, and intraspecies), the degrees of variability of the intron markers were all approximately twofold greater than that of the NPC markers (Figure 2). Figure S2 further illustrates the genetic distance for each of the 96 markers at four different taxonomic levels (details can be found in Table S1). The average *p‐*distances of the intron markers at these four levels were .168, .052, .015, and .004, respectively. In general, the evolutionary rate analyses indicated that our intron markers were informative and had the potential to resolve both deep and shallow‐scale snake phylogenetic questions.

**Figure 3:**
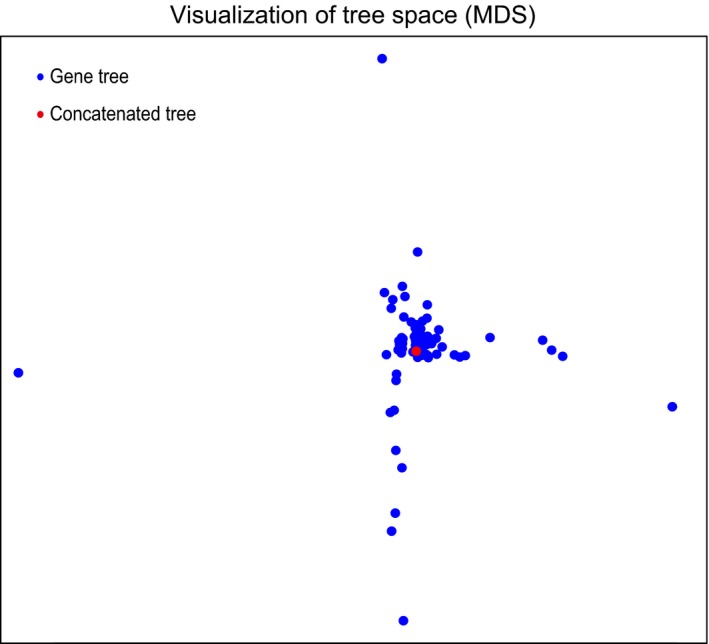
Visualization of tree space using multidimensional scaling (MDS) for the gene trees generated from the intron markers and the concatenated Maximum‐likelihood (ML) tree. Blue points represent the ML gene tree from each of the 96 markers, while the red point indicates the tree from concatenation analysis using RAxML with 96 partitions

In addition, to assess how close the tree topology of each marker was to each other and to the concatenated data set, unweighted RF distances of individual gene trees and the concatenated tree were calculated and visualized in an MDS plot (Figure 3). Our results showed that the tree space occupied by the majority of the intron markers was very close or largely overlapped with each other. They formed an “island” in the MDS plot, and the tree space occupied by the reference topology (the concatenated tree from RaxML) was located within the “island.” The deviating points were mostly markers that had very low sequence recovery rates. It suggested that the phylogenetic signals were generally congruent within the intron markers.

### 3.3 Robust snake phylogenies were inferred from the intron markers

**Figure 4:**
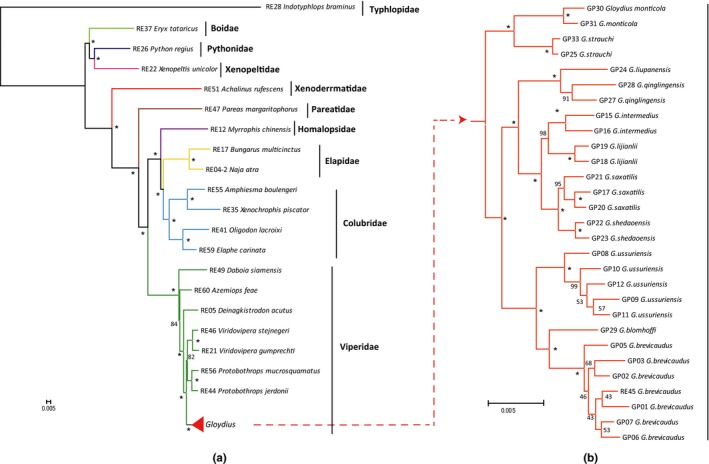
Phylogeny of 49 snakes inferred from the 96 intron markers. The tree was inferred by concatenation analysis using RAxML. For better display, it is shown in two parts with different scale bar. Part (a) displays the family‐level phylogeny, while part (b) exhibits the relationships within the genus *Gloydius*. Bootstrap supports for each branch are shown close to node and an asterisk indicates support =100

With a dataset of 96 intron markers comprising a total of 86,826 base pairs, we reconstructed the snake phylogeny. The phylogenetic tree obtained from concatenated likelihood analysis (RAxML) (Figure 4) was generally highly robust. In all 46 nodes of the tree, 33 had statistical support of 100%, and the mean support value was 91.5%. The phylogenetic relationship derived from the species‐tree (ASTRAL) method produced highly similar topology to the concatenated analysis with high supports for the majority of the nodes (Fig. S1).

At higher level, the family‐wide relationships were all strongly supported (Figure 4 and S1) and largely concordant with those from recent molecular phylogenetic studies of other groups (Pyron et al., 2013, 2014; Streicher & Wiens, 2016; Wiens et al., 2012; Zheng & Wiens, 2015). At the base of the snake phylogeny, a clade uniting Pythonidae and Xenopeltidae was sister to Boidae. Within caenophidians, xenodermatids was sister to all other caenophidians, followed successively by pareatids, viperids, homalopsids, elapids, and the colubrids. The relationship within Viperidae strongly placed Viperinae as sister to the other two subfamilies (Crotalinae and Azemiopinae).

At the shallow scale, the phylogenetic relationship of *Gloydius* (Serpentes: Crotalinae) (Figure 4) was also well resolved. The base of the phylogeny was a clade uniting *G. strauchi* and *G. monticola*, which are the two montane species from southwest China. The remaining *Gloydius* were divided into two strongly supported clades. Our analysis clearly showed that *G. liupanensis* and *G. qinglingensis* formed a clade closely related to *G. intermedius*,* G. lijianlii*,* G. saxatilis,* and *G. shedaoensis*, but far from *G. strauchi* and *G. monticola*. Thus, *G. liupanensis* and *G. qinglingensis* were not subspecies or populations of *G. strauchi* (Wang & Zhao, 2006), and the montane species were not one monophyletic group. For the two insular endemic species, our analysis indicated that *G. lijianlii* (from the northern coastal islands along Shandong Peninsula) was closely related to *G. intermedius*, which were sampled in Shaanxi Province in north China, while *G. shedaoensis* (restricted to the Snake Island in Northeastern China) was closely related to *G. saxatilis*, which were collected in Liaoning and Jilin provinces in Northeastern China. Thus, the insular species in the Bohai area were not a special clade, but probably spread from their nearest mainland area, respectively. Within the other clade, *G. brevicaudus* showed a sister relationship with *G. blomhoffi*, and then both were sisterly related to *G. ussuriensis*. This relationship was similar to that from the mitochondrial markers (Xu et al., 2012). At shallower level, our intron markers showed some degree of information for relationships within *G. ussuriensis* and *G. brevicaudus* (Figure 4). Relationships estimated by ASTRAL (Fig. S1) were largely similar to those estimated by RAxML with only slight differences in subspecies arrangements. These results demonstrated that our newly developed intron markers had sufficient phylogenetic information to resolve interspecific relationships of snakes and were also helpful at intraspecific level.

## 4 DISCUSSION

In the search for molecular markers to resolve phylogenetic questions, intron sequences are receiving an increasing amount of attention because of their elevated evolutionary rate, which provides considerable phylogenetic signal, and their low level of functional constraint, which reduces gene tree incongruence (Dool et al., 2016; Foley et al., 2015; Jarvis et al., 2014; Yu et al., 2011). The development of intron markers, nevertheless, for a long time lagged behind coding markers, as it requires genomic sequences and more analytical considerations. The extensive application of intron markers is hindered by issues including optimizing PCR, sequencing of a large number of amplicons, alignment of diverged sequences, etc. In this study, we applied an improved and efficient data mining strategy to two snake genome sequences which identified appropriate introns between single‐copy and conserved exon pairs with constraints on sequence length variability and sequence variability. Over a thousand of potential intron loci were retrieved, and 96 novel intron markers were developed. By applying nested PCR, the markers could be amplified across a wide range of snake taxa with no need of further optimization and yielded fairly high PCR successful rates. Most of the PCR reactions produced a single band, indicating that the upstream screening of single‐copy exons was effective. The analysis in a real case demonstrated that the newly developed intron markers were more informative than coding markers and performed well in both deep and shallow evolutionary depths. They gave a clear phylogeny of the *Gloydius* genus, the basal clade of which including two species from the mountains of southwest China is consistent with the hypothesis that the region of Hengduan Mountains was an origin center for many Asian animals (Zhao & Yang, 1997). Moreover, the intron markers clarified the relationships of the montane and insular *Gloydius* snakes, which were not revealed by previous studies with other type of markers (Xu et al., 2012). Thus, this suite of novel intron markers is very likely to shed new light on the contentious nodes in the snake tree, and may also be useful in species delimitation. In addition, from the candidate pool, more markers can be readily developed through quick screening and testing to serve different purposes, such as to increase the data amount or to study a specific phylogenetic question. They will be a powerful tool toward fully resolving the snake tree.

Intron markers provide a convenient way to obtain the phylogenetic signals in noncoding genomic regions. It is a good addition to the ANMs and the UCE capture approach. The development of ANMs involves extensive cloning and sequencing of the clone inserts, thus the marker number was difficult to scale‐up. It also takes effort to detect and avoid repetitive elements during ANM development. In addition, the application range of ANMs was very restricted, due to high mutation rates in priming sites (Thomson, Shedlock, Edwards, & Shaffer, 2008; Thomson et al., 2010). The superior advantage of the sequence capture approach is well known, which can simultaneously produce hundreds to thousands of loci for tens of individuals within a relatively short time and can be very cost‐effective (Faircloth et al., 2012; Lemmon, Emme, & Lemmon, 2012; McCormack et al., 2012; Prum et al., 2015). However, downstream sequence processing is a challenge to many researchers because the phylogenetic signals are not within the UCE probes but in their flanking sequences which are not certain in length and may involve much effort in distinguishing misassembled sequences from the true sequences. Intron markers rely on traditional PCR and are able to generate medium‐scale multilocus data, which are informative to produce high‐resolution phylogenies in most cases due to upstream bioinformatic screening (Dool et al., 2016; Egea et al., 2016; Igea et al., 2010; Yu & Zhang, 2006; Yu et al., 2011). With the help of the adjacent exons and the use of nested PCR, the experimental successful rate of intron markers is generally high, which means lower rate of missing data as compared with the captured sequences. The adjacent exons set the boundaries for intron markers, and also make orthology determination and sequence alignment relatively easy for intron marker sequences. Currently, PCR products can be subjected to NGS, as has been done in this and several other studies (Che et al., 2017; Feng et al., 2016). Obtaining reliable intron marker sequences from the assembled contigs, though not as straightforward as the mitochondrial and NPC markers, has been shown to be viable with more analytical criteria considering sequence boundaries and against chimeras. Allele differences are common for introns. Intraindividual allele heterozygotes generally form monophyletic pairs on the phylogenetic tree (Yu et al., 2011). Thus, we used consensus sequences to study the snake relationships at and above species level. Further analyses focusing on SNPs and strategies to obtain the intraindividual alleles will reveal more information about the intraspecific divergences and the recent histories of these species. NGS skips the laborious and time‐consuming gel cutting and Sanger sequencing steps, thus shortens the time and lowers the cost for obtaining a large number of marker sequences from many different samples. Therefore, sequencing is no longer a big burden for the PCR‐based method. If combined with the promising high‐throughput PCR techniques such as multiplex PCR or microdroplet PCR to accelerate the amplification step, the PCR‐based method will be even easier and faster. In the future, molecular markers, with the incorporation of the state‐of‐the‐art approaches, will continue playing important roles in the field of phylogenetics, among which intron markers are most promising and deserve more attention and further exploration.

## 5 CONCLUSION

We used snakes as a model to pave the way for massive identification and application of intron markers. By scanning the snake genomes with an improved and effective bioinformatic pipeline, we retrieved over a thousand candidate intron loci, then developed 96 novel intron markers. The use of nested PCR generates high PCR successful rates of the markers across a wide range of snake taxa. The large number of amplicon sequences was readily recovered from NGS following the appropriate sequence processing steps. The intron markers were demonstrated to be useful in reconstructing the snake relationships from family to species level. This suite of intron markers will be an effective tool for the molecular phylogenetic studies of snakes.

## CONFLICT OF INTEREST

None declared.

## AUTHOR CONTRIBUTIONS

PZ and DL conceived the ideas and designed methodology; CH wrote the python scripts for data mining; PG provided all the *Gloydius* samples; JNL and CH performed the experiment and analyzed the data; DL and JNL led the writing of the manuscript. All authors contributed critically to the drafts and gave final approval for publication.

## DATA ACCESSIBILITY

NEXUS alignments for phylogenetic analyses and the candidate database of our newly developed intron markers (nucleotide sequence of the 1,273 marker candidates) are available from Figshare Repository. https://figshare.com/s/5f51ee129ef810898daa
