# Supplementary material for: A workflow of massive identification and application of intron markers using snakes as a model
Source: Ecol Evol. 2017 Oct 22;7(23):10042–55. doi: 10.1002/ece3.3525 (PMC5723593; doi:10.1002/ece3.3525)
Supplement: Supplementary file 1 [file ECE3-7-10042-s001.pdf]

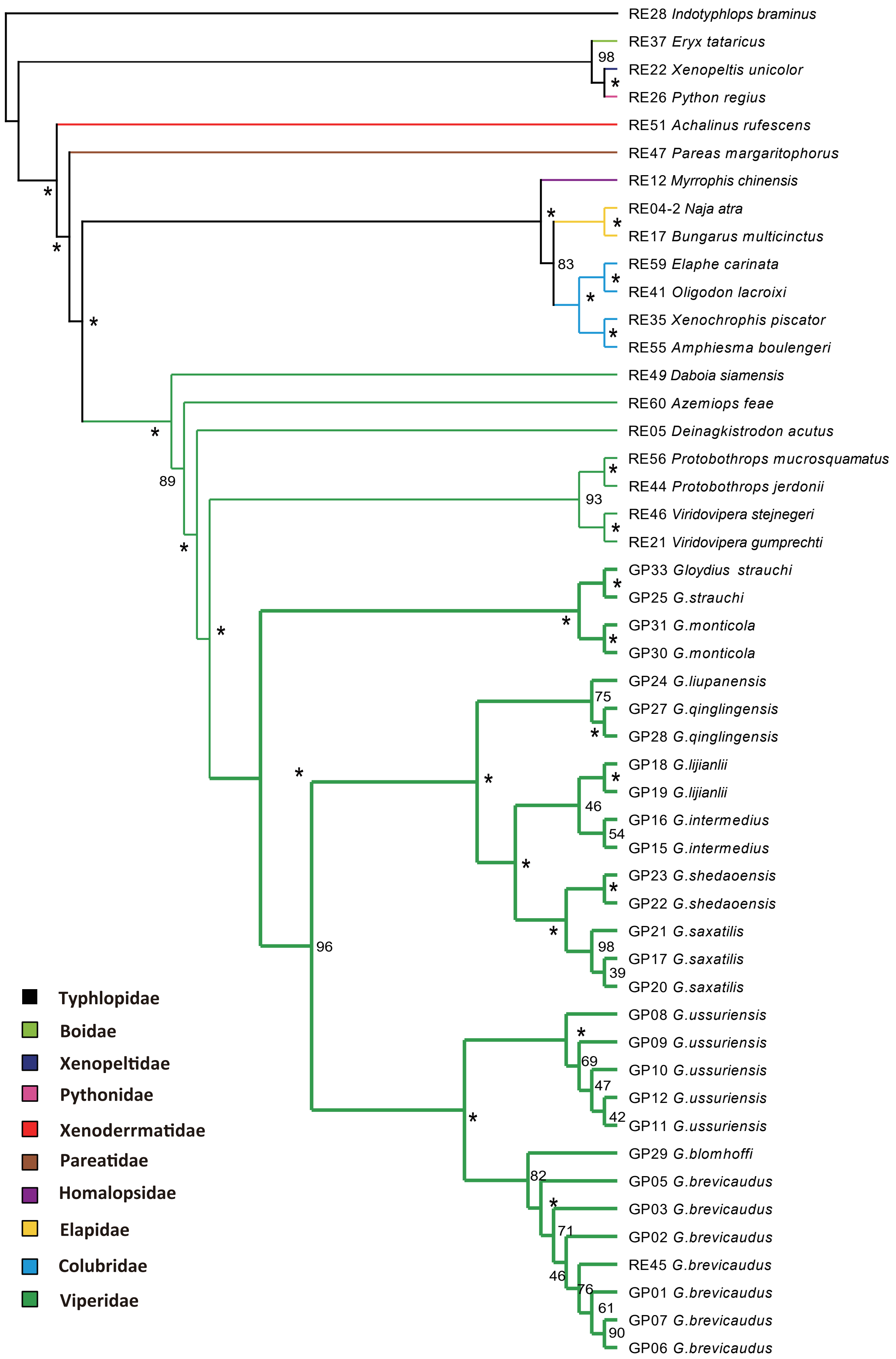


**Fig. S1** Species-tree analysis (ASTRAL) resulting from the analysis of the 96 intron markers. Branches are colored according to the families. The genus *Gloydius* (Viperidae) is indicated with bold lines. Numbers adjacent to the nodes are support values and an asterisk indicates support = 100.

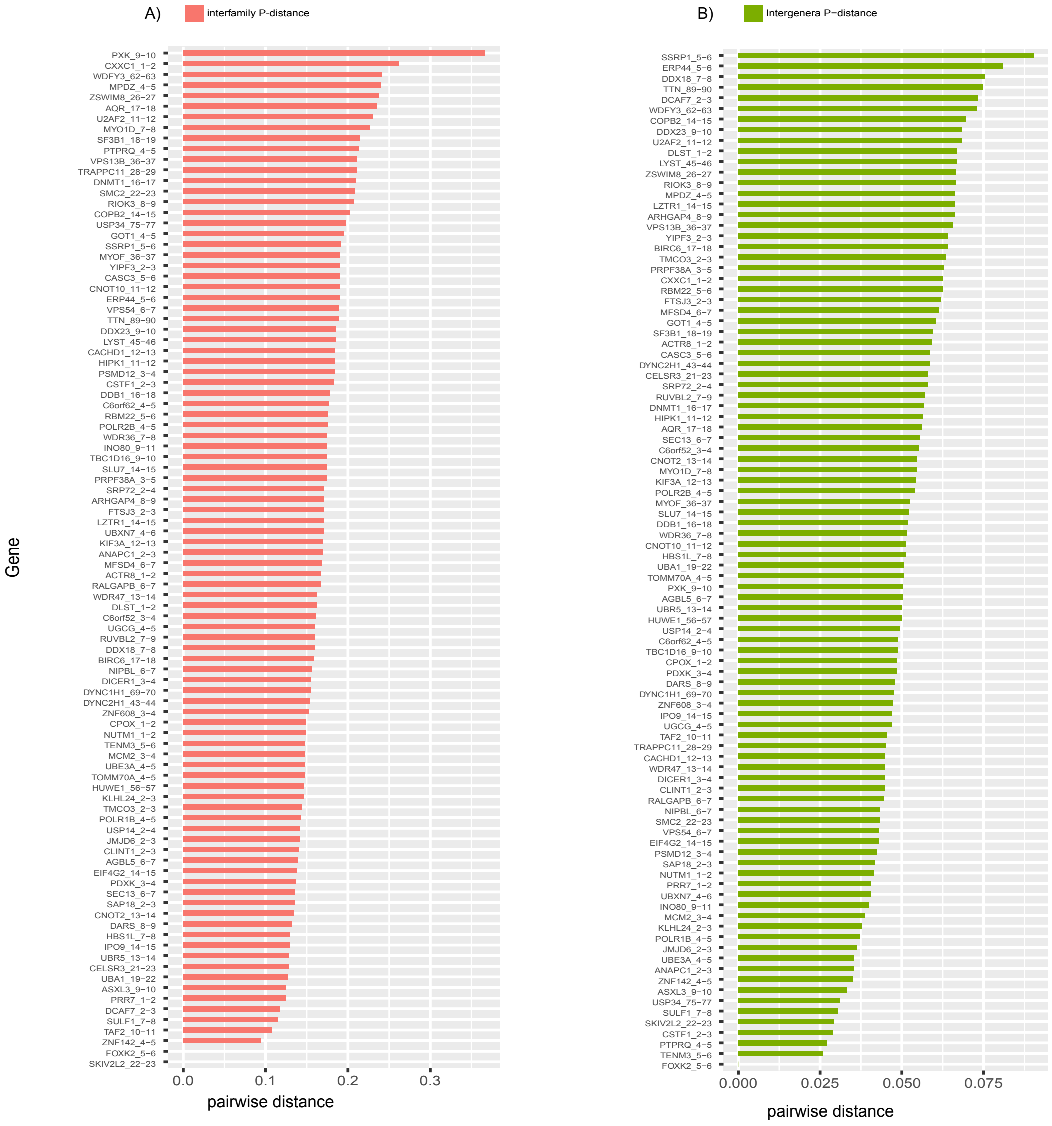

A)
interfamily P-distance
Gene
PXK_9−10
CXXC1_1−2
WDFY3_62−63
MPDZ_4−5
ZSWIM8_26−27
AQR_17−18
U2AF2_11−12
MYO1D_7−8
SF3B1_18−19
PTPRQ_4−5
VPS13B_36−37
TRAPPC11_28−29
DNMT1_16−17
SMC2_22−23
RIOK3_8−9
COPB2_14−15
USP34_75−77
GOT1_4−5
SSRP1_5−6
MYOF_36−37
YIPF3_2−3
CASC3_5−6
CNOT10_11−12
ERP44_5−6
VPS54_6−7
TTN_89−90
DDX23_9−10
LYST_45−46
CACHD1_12−13
HIPK1_11−12
PSMD12_3−4
CSTF1_2−3
DDB1_16−18
C6orf62_4−5
RBM22_5−6
POLR2B_4−5
WDR36_7−8
INO80_9−11
TBC1D16_9−10
SLU7_14−15
PRPF38A_3−5
SRP72_2−4
ARHGAP4_8−9
FTSJ3_2−3
LZTR1_14−15
UBXN7_4−6
KIF3A_12−13
ANAPC1_2−3
MFSD4_6−7
ACTR8_1−2
RALGAPB_6−7
WDR47_13−14
DLST_1−2
C6orf52_3−4
UGCG_4−5
RUVBL2_7−9
DDX18_7−8
BIRC6_17−18
NIPBL_6−7
DICER1_3−4
DYNC1H1_69−70
DYNC2H1_43−44
ZNF608_3−4
CPOX_1−2
NUTM1_1−2
TENM3_5−6
MCM2_3−4
UBE3A_4−5
TOMM70A_4−5
HUWE1_56−57
KLHL24_2−3
TMCO3_2−3
POLR1B_4−5
USP14_2−4
JMJD6_2−3
CLINT1_2−3
AGBL5_6−7
EIF4G2_14−15
PDXK_3−4
SEC13_6−7
SAP18_2−3
CNOT2_13−14
DARS_8−9
HBS1L_7−8
IPO9_14−15
UBR5_13−14
CELSR3_21−23
UBA1_19−22
ASXL3_9−10
PRR7_1−2
DCAF7_2−3
SULF1_7−8
TAF2_10−11
ZNF142_4−5
FOXK2_5−6
SKIV2L2_22−23
0.0
0.1
0.2
0.3
pairwise distance
B)
Intergenera P−distance
SSRP1_5−6
ERP44_5−6
DDX18_7−8
TTN_89−90
DCAF7_2−3
WDFY3_62−63
COPB2_14−15
DDX23_9−10
U2AF2_11−12
DLST_1−2
LYST_45−46
ZSWIM8_26−27
RIOK3_8−9
MPDZ_4−5
LZTR1_14−15
ARHGAP4_8−9
VPS13B_36−37
YIPF3_2−3
BIRC6_17−18
TMCO3_2−3
PRPF38A_3−5
CXXC1_1−2
RBM22_5−6
FTSJ3_2−3
MFSD4_6−7
GOT1_4−5
SF3B1_18−19
ACTR8_1−2
CASC3_5−6
DYNC2H1_43−44
CELSR3_21−23
SRP72_2−4
RUVBL2_7−9
DNMT1_16−17
HIPK1_11−12
AQR_17−18
SEC13_6−7
C6orf52_3−4
CNOT2_13−14
MYO1D_7−8
KIF3A_12−13
POLR2B_4−5
MYOF_36−37
SLU7_14−15
DDB1_16−18
WDR36_7−8
CNOT10_11−12
HBS1L_7−8
UBA1_19−22
TOMM70A_4−5
PXK_9−10
AGBL5_6−7
UBR5_13−14
HUWE1_56−57
USP14_2−4
C6orf62_4−5
TBC1D16_9−10
CPOX_1−2
PDXK_3−4
DARS_8−9
DYNC1H1_69−70
ZNF608_3−4
IPO9_14−15
UGCG_4−5
TAF2_10−11
TRAPPC11_28−29
CACHD1_12−13
WDR47_13−14
DICER1_3−4
CLINT1_2−3
RALGAPB_6−7
NIPBL_6−7
SMC2_22−23
VPS54_6−7
EIF4G2_14−15
PSMD12_3−4
SAP18_2−3
NUTM1_1−2
PRR7_1−2
UBXN7_4−6
INO80_9−11
MCM2_3−4
KLHL24_2−3
POLR1B_4−5
JMJD6_2−3
UBE3A_4−5
ANAPC1_2−3
ZNF142_4−5
ASXL3_9−10
USP34_75−77
SULF1_7−8
SKIV2L2_22−23
CSTF1_2−3
PTPRQ_4−5
TENM3_5−6
FOXK2_5−6
0.000
0.025
0.050
0.075
pairwise distance

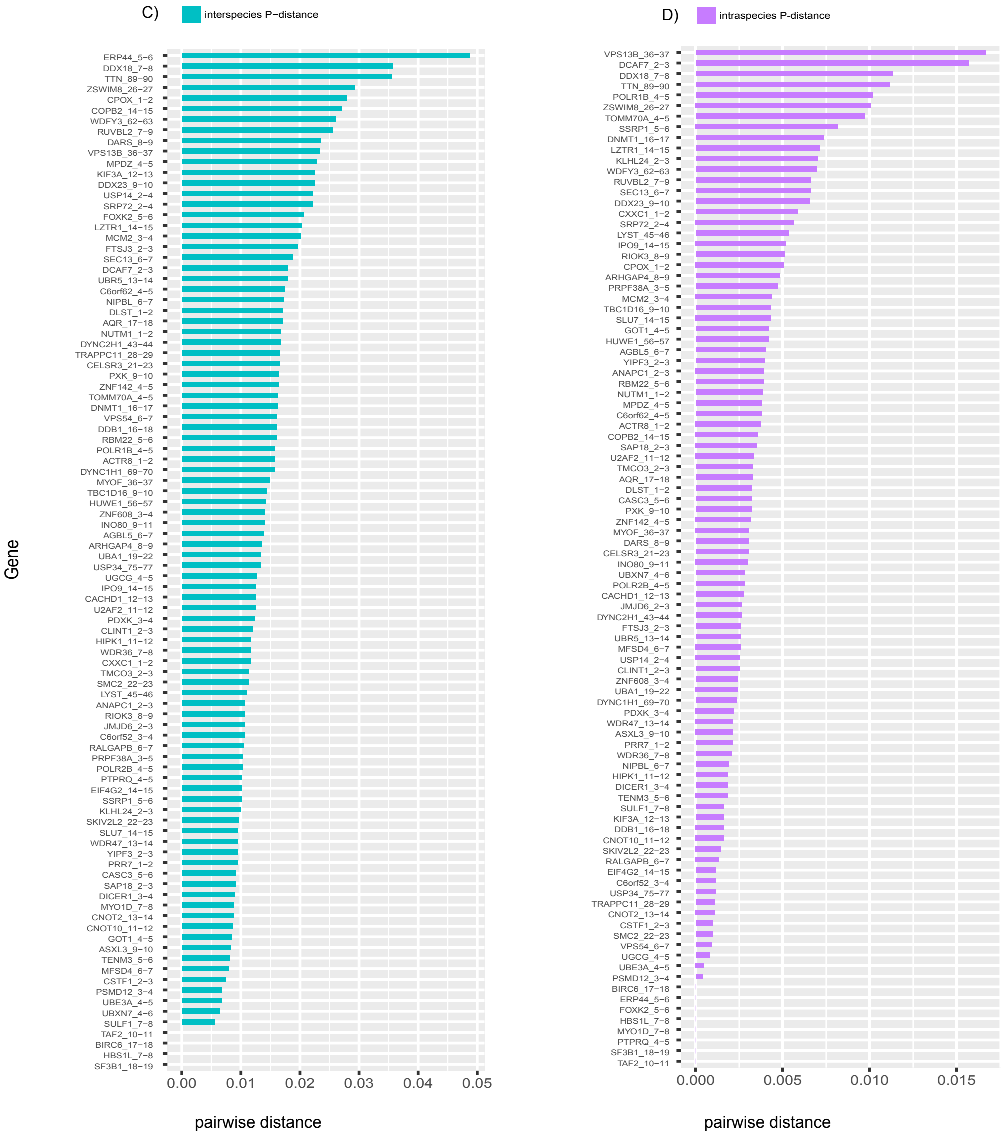


**Fig. S2** Sequence divergences of the 96 intron markers at different taxonomic levels. The average p-distances for each nuclear locus are estimated at four levels: A. interfamily (species in different family), B. intergenus (species in the same family but different genera), C. interspecies and D. intraspecies.

**Table S1: The average p-distances at four taxonomic levels of the 96 novel intron markers.**

| Loci | Interfamily P-distance | Intergenera P-distance | Interspecies P-distance | Intraspecies P-distance |
|---|---|---|---|---|
| ACTR8_1-2 | 0.1676 | 0.0592 | 0.0157 | 0.0037 |
| AGBL5_6-7 | 0.1397 | 0.0503 | 0.0139 | 0.0040 |
| ANAPC1_2-3 | 0.1692 | 0.0353 | 0.0107 | 0.0039 |
| AQR_17-18 | 0.2349 | 0.0561 | 0.0171 | 0.0033 |
| ARHGAP4_8-9 | 0.1710 | 0.0661 | 0.0135 | 0.0048 |
| ASXL3_9-10 | 0.1251 | 0.0332 | 0.0083 | 0.0021 |
| BIRC6_17-18 | 0.1588 | 0.0640 | --- | --- |
| C6orf52_3-4 | 0.1610 | 0.0551 | 0.0106 | 0.0012 |
| C6orf62_4-5 | 0.1766 | 0.0489 | 0.0175 | 0.0038 |
| CACHD1_12-13 | 0.1842 | 0.0449 | 0.0126 | 0.0028 |
| CASC3_5-6 | 0.1903 | 0.0586 | 0.0092 | 0.0032 |
| CELSR3_21-23 | 0.1276 | 0.0579 | 0.0166 | 0.0030 |
| CLINT1_2-3 | 0.1401 | 0.0447 | 0.0121 | 0.0025 |
| CNOT10_11-12 | 0.1901 | 0.0512 | 0.0087 | 0.0016 |
| CNOT2_13-14 | 0.1339 | 0.0546 | 0.0088 | 0.0011 |
| COPB2_14-15 | 0.2028 | 0.0697 | 0.0271 | 0.0036 |
| CPOX_1-2 | 0.1491 | 0.0486 | 0.0279 | 0.0051 |
| CSTF1_2-3 | 0.1832 | 0.0288 | 0.0074 | 0.0010 |
| CXXC1_1-2 | 0.2621 | 0.0626 | 0.0116 | 0.0059 |
| DARS_8-9 | 0.1313 | 0.0479 | 0.0236 | 0.0030 |
| DCAF7_2-3 | 0.1174 | 0.0733 | 0.0179 | 0.0157 |
| DDB1_16-18 | 0.1779 | 0.0517 | 0.0160 | 0.0016 |
| DDX18_7-8 | 0.1592 | 0.0753 | 0.0358 | 0.0113 |
| DDX23_9-10 | 0.1857 | 0.0684 | 0.0225 | 0.0066 |
| DICER1_3-4 | 0.1551 | 0.0449 | 0.0089 | 0.0019 |
| DLST_1-2 | 0.1617 | 0.0669 | 0.0171 | 0.0032 |
| DNMT1_16-17 | 0.2096 | 0.0568 | 0.0163 | 0.0074 |
| DYNC1H1_69-70 | 0.1544 | 0.0475 | 0.0157 | 0.0024 |
| DYNC2H1_43-44 | 0.1540 | 0.0584 | 0.0167 | 0.0026 |
| EIF4G2_14-15 | 0.1376 | 0.0429 | 0.0102 | 0.0012 |
| ERP44_5-6 | 0.1898 | 0.0809 | 0.0488 | --- |
| FOXK2_5-6 | --- | --- | 0.0207 | --- |
| FTSJ3_2-3 | 0.1705 | 0.0618 | 0.0197 | 0.0026 |
| GOT1_4-5 | 0.1946 | 0.0603 | 0.0085 | 0.0042 |
| HBS1L_7-8 | 0.1299 | 0.0511 | --- | --- |
| HIPK1_11-12 | 0.1841 | 0.0564 | 0.0117 | 0.0019 |
| HUWE1_56-57 | 0.1469 | 0.0501 | 0.0142 | 0.0042 |
| INO80_9-11 | 0.1744 | 0.0398 | 0.0141 | 0.0030 |
| IPO9_14-15 | 0.1292 | 0.0470 | 0.0126 | 0.0052 |
| JMJD6_2-3 | 0.1412 | 0.0363 | 0.0107 | 0.0026 |
| KIF3A_12-13 | 0.1699 | 0.0544 | 0.0225 | 0.0016 |
| KLHL24_2-3 | 0.1461 | 0.0377 | 0.0100 | 0.0070 |
| LYST_45-46 | 0.1847 | 0.0668 | 0.0110 | 0.0054 |
| LZTR1_14-15 | 0.1703 | 0.0661 | 0.0203 | 0.0071 |
| MCM2_3-4 | 0.1475 | 0.0387 | 0.0201 | 0.0044 |
| MFSD4_6-7 | 0.1683 | 0.0613 | 0.0079 | 0.0026 |
| MPDZ_4-5 | 0.2397 | 0.0663 | 0.0228 | 0.0038 |
| MYO1D_7-8 | 0.2265 | 0.0546 | 0.0088 | --- |
| MYOF_36-37 | 0.1904 | 0.0525 | 0.0149 | 0.0031 |
| NIPBL_6-7 | 0.1557 | 0.0433 | 0.0173 | 0.0019 |
| NUTM1_1-2 | 0.1489 | 0.0415 | 0.0168 | 0.0038 |

| | | | | |
|---|---|---|---|---|
| PDXK_3-4 | 0.1368 | 0.0483 | 0.0123 | 0.0022 |
| POLR1B_4-5 | 0.1427 | 0.0370 | 0.0158 | 0.0102 |
| POLR2B_4-5 | 0.1750 | 0.0539 | 0.0104 | 0.0028 |
| PRPF38A_3-5 | 0.1738 | 0.0629 | 0.0104 | 0.0047 |
| PRR7_1-2 | 0.1245 | 0.0405 | 0.0094 | 0.0021 |
| PSMD12_3-4 | 0.1838 | 0.0424 | 0.0068 | 0.0004 |
| PTPRQ_4-5 | 0.2127 | 0.0271 | 0.0102 | --- |
| PXK_9-10 | 0.3662 | 0.0503 | 0.0165 | 0.0032 |
| RALGAPB_6-7 | 0.1669 | 0.0445 | 0.0105 | 0.0014 |
| RBM22_5-6 | 0.1761 | 0.0624 | 0.0160 | 0.0039 |
| RIOK3_8-9 | 0.2077 | 0.0664 | 0.0107 | 0.0051 |
| RUVBL2_7-9 | 0.1596 | 0.0569 | 0.0255 | 0.0066 |
| SAP18_2-3 | 0.1350 | 0.0417 | 0.0091 | 0.0035 |
| SEC13_6-7 | 0.1355 | 0.0554 | 0.0188 | 0.0066 |
| SF3B1_18-19 | 0.2144 | 0.0595 | --- | --- |
| SKIV2L2_22-23 | --- | 0.0292 | 0.0097 | 0.0014 |
| SLU7_14-15 | 0.1739 | 0.0522 | 0.0095 | 0.0043 |
| SMC2_22-23 | 0.2084 | 0.0433 | 0.0113 | 0.0010 |
| SRP72_2-4 | 0.1712 | 0.0578 | 0.0221 | 0.0056 |
| SSRP1_5-6 | 0.1918 | 0.0903 | 0.0101 | 0.0082 |
| SULF1_7-8 | 0.1151 | 0.0303 | 0.0056 | 0.0016 |
| TAF2_10-11 | 0.1073 | 0.0453 | --- | --- |
| TBC1D16_9-10 | 0.1744 | 0.0487 | 0.0144 | 0.0043 |
| TENM3_5-6 | 0.1479 | 0.0258 | 0.0082 | 0.0018 |
| TMCO3_2-3 | 0.1443 | 0.0633 | 0.0113 | 0.0033 |
| TOMM70A_4-5 | 0.1471 | 0.0505 | 0.0163 | 0.0097 |
| TRAPPC11_28-29 | 0.2104 | 0.0451 | 0.0166 | 0.0011 |
| TTN_89-90 | 0.1884 | 0.0748 | 0.0355 | 0.0111 |
| U2AF2_11-12 | 0.2297 | 0.0684 | 0.0125 | 0.0033 |
| UBA1_19-22 | 0.1267 | 0.0506 | 0.0134 | 0.0024 |
| UBE3A_4-5 | 0.1474 | 0.0354 | 0.0067 | 0.0005 |
| UBR5_13-14 | 0.1277 | 0.0501 | 0.0179 | 0.0026 |
| UBXN7_4-6 | 0.1701 | 0.0405 | 0.0064 | 0.0028 |
| UGCG_4-5 | 0.1598 | 0.0468 | 0.0127 | 0.0008 |
| USP14_2-4 | 0.1414 | 0.0495 | 0.0222 | 0.0026 |
| USP34_75-77 | 0.1980 | 0.0309 | 0.0133 | 0.0012 |
| VPS13B_36-37 | 0.2113 | 0.0656 | 0.0233 | 0.0167 |
| VPS54_6-7 | 0.1890 | 0.0429 | 0.0161 | 0.0009 |
| WDFY3_62-63 | 0.2408 | 0.0730 | 0.0260 | 0.0069 |
| WDR36_7-8 | 0.1748 | 0.0514 | 0.0116 | 0.0021 |
| WDR47_13-14 | 0.1625 | 0.0449 | 0.0095 | 0.0022 |
| YIPF3_2-3 | 0.1904 | 0.0641 | 0.0094 | 0.0040 |
| ZNF142_4-5 | 0.0945 | 0.0351 | 0.0164 | 0.0032 |
| ZNF608_3-4 | 0.1522 | 0.0471 | 0.0141 | 0.0024 |
| ZSWIM8_26-27 | 0.2373 | 0.0665 | 0.0293 | 0.0101 |

"--" means not applicable due to lack of data.

## Table S2 Nested PCR primer sequences for the 96 novel intron markers.

| ID | Gene | Primer | Assay | Nucleic acid sequence (5'-3') | GC(%) | Tm | *Product size (bp)≈ | PCR success rate in 16 test vertebrates |
|---|---|---|---|---|---|---|---|---|
| 1 | ACTR8 | ACTR8_1-2_F1 | 1st PCR | TGRTADATCCANARYTCYTGNGT | 40.5 | 57.6 | 1098 | 15(93%) |
| | | ACTR8_1-2_R1 | | ATGTTYCAYAARGCNCARGARTT | 39.1 | 57 | | |
| | | ACTR8_1-2_F2 | 2nd PCR | ACNGCNCCNCCYTTCCANGCDAT | 60.1 | 65.6 | | |
| | | ACTR8_1-2_R2 | | GTNGARAAYGTNGARGTNATHAC | 40.5 | 57.6 | | |
| 2 | AGBL5 | AGBL5_6-7_F1 | 1st PCR | TCYTTNGCRTACATRTTYTTYTC | 34.7 | 55.3 | 710 | 16(100%) |
| | | AGBL5_6-7_R1 | | TAYGTYGAYYTNCAYGGNCAYGC | 52.1 | 62.4 | | |
| | | AGBL5_6-7_F2 | 2nd PCR | TTNGGRAANARCATRTTYTCNAC | 36.9 | 56.1 | | |
| | | AGBL5_6-7_R2 | | TGYTTYATGTAYGGNAAYAAYAT | 30.4 | 53.5 | | |
| 3 | ANAPC1 | ANAPC1_2-3_F1 | 1st PCR | GAYCAYTGYAARCAYCAYCCNAA | 45.6 | 59.7 | 1294 | 16(100%) |
| | | ANAPC1_2-3_R1 | | TCNACNGTRAANGCYTTRTA | 40 | 53.2 | | |
| | | ANAPC1_2-3_F2 | 2nd PCR | CARGARGTNACNATHCAYGARAA | 40.5 | 57.6 | | |
| | | ANAPC1_2-3_R2 | | TCYTCRTCRTAYTCNACYTCYTC | 45.6 | 59.7 | | |
| 4 | AQR | AQR_17-18_F1 | 1st PCR | CKCATDATDATRTTRAANGTYTC | 31.1 | 53.8 | 1450 | 16(100%) |
| | | AQR_17-18_R1 | | CARCCNTTYGTNGARCARACNGG | 54.3 | 63.3 | | |
| | | AQR_17-18_F2 | 2nd PCR | TCYTGYTGRTAYTGRTTNGGRTC | 45.6 | 59.7 | | |
| | | AQR_17-18_R2 | | GGNTGYGARATHCARGGNATGYT | 49.2 | 61.2 | | |
| 5 | ARHGAP4 | ARHGAP4_8-9_F1 | 1st PCR | TCNCCYTTYTCDATNGCYTCYTT | 44.9 | 59.4 | 1376 | 16(100%) |
| | | ARHGAP4_8-9_R1 | | GAYGCNTTYCARGGNTGYCA | 55 | 59.3 | | |
| | | ARHGAP4_8-9_F2 | 2nd PCR | TGRTCRTGYTTNGCYTGNARYTT | 43.4 | 58.8 | | |
| | | ARHGAP4_8-9_R2 | | GAYGCNGGNGGNAARCARGC | 65 | 63.4 | | |
| 6 | ASXL3 | ASXL3_9-10_F1 | 1st PCR | TCNCCRTTYTCYTCNCCRTT | 50 | 57.3 | 1298 | 15(93%) |
| | | ASXL3_9-10_R1 | | AAYGCNATGYTNCAYACNAAYAC | 41.3 | 57.9 | | |
| | | ASXL3_9-10_F2 | 2nd PCR | GTNCCRTCNGCNGGRCANGT | 65 | 63.4 | | |
| | | ASXL3_9-10_R2 | | TTYTTYAARATHCCNGGNAARGC | 40.5 | 57.6 | | |
| 7 | BIRC6 | BIRC6_17-18_F1 | 1st PCR | ACNSWRTARTTRTCYTGRAANCC | 41.3 | 57.9 | 1148 | 14(87%) |
| | | BIRC6_17-18_R1 | | TAYAARGGNMGNATHAAYGC | 41.6 | 53.8 | | |
| | | BIRC6_17-18_F2 | 2nd PCR | TTYTCYTTRTCYTCYTTYTGYTC | 36.9 | 56.1 | | |
| | | BIRC6_17-18_R2 | | ATHCARCAYCCNATGTAYGGNGC | 51.4 | 62.1 | | |
| 8 | C6orf52 | C6orf52_3-4_F1 | 1st PCR | TCRTTNCKRTCRTARTTRTAYTG | 34.7 | 55.3 | 1248 | 14(87%) |
| | | C6orf52_3-4_R1 | | WSNGAYGARGGNGAYATGATGMG | 52.1 | 62.4 | | |
| | | C6orf52_3-4_F2 | 2nd PCR | TGRTARTCYTGRTARTARTCRTT | 32.6 | 54.4 | | |
| | | C6orf52_3-4_R2 | | CARGAYTGYCARAAYGCNCCNGG | 58.6 | 65.1 | | |
| 9 | C6orf62 | C6orf62_4-5_F1 | 1st PCR | CAYCAYGGNGAYTAYGARAARCA | 45.6 | 59.7 | 1150 | 16(100%) |
| | | C6orf62_4-5_R1 | | TCYGGCATRTANGGNCKNARRTG | 52.1 | 62.4 | | |
| | | C6orf62_4-5_F2 | 2nd PCR | ATHGTNATHAAYAAYCCNAAYCA | 31.1 | 53.8 | | |
| | | C6orf62_4-5_R2 | | CCARTGRGTNARYTGYTCYTGNGG | 56.2 | 65.2 | | |
| 10 | CACHD1 | CACHD1_12-13_F1 | 1st PCR | TANGGNCCNGTRAANGTDAT | 44.1 | 54.9 | 1373 | 16(100%) |
| | | CACHD1_12-13_R1 | | GTNMGNAAYGARGTNATGGCNAC | 50 | 61.5 | | |
| | | CACHD1_12-13_F2 | 2nd PCR | CCNGGRTTNGCNACNGCRTG | 65 | 63.4 | | |
| | | CACHD1_12-13_R2 | | ATGGAYAARGCNTTYGAYCCNAC | 47.8 | 60.6 | | |

| | | | | | | | | |
|---|---|---|---|---|---|---|---|---|
| 11 | CASC3 | CASC3_5-6_F1 | 1st PCR | AARGGNYTNTTYTTYGARCAYGA | 39.1 | 57 | 923 | 15(93%) |
| | | CASC3_5-6_R1 | | GCYTGYTCRTCYTCNCKRAAYTT | 47.8 | 60.6 | | |
| | | CASC3_5-6_F2 | 2nd PCR | GGNCARACNCARGARGARGARGT | 54.3 | 63.3 | | |
| | | CASC3_5-6_R2 | | TGYTCCCANCKNCCYTCRTCYTT | 54.3 | 63.3 | | |
| 13 | CELSR3 | CELSR3_21-23_F1 | 1st PCR | GAYMGNATGGARAAYCAYACNCA | 45.6 | 59.7 | 1263 | 16(100%) |
| | | CELSR3_21-23_R1 | | TGRTTCCAYTGNACRCANAGNGG | 52.1 | 62.4 | | |
| | | CELSR3_21-23_F2 | 2nd PCR | TTYMGNGGNCARGCNATGTGGGA | 56.5 | 64.2 | | |
| | | CELSR3_21-23_R2 | | ACDATGGGNSWRTTCATNAC | 39.2 | 54.9 | | |
| 14 | CLINT1 | CLINT1_2-3_F1 | 1st PCR | ATCATWGTGTTSARNSWNGGYTG | 43.4 | 58.8 | 1321 | 15(93%) |
| | | CLINT1_2-3_R1 | | GGWGARTTYGGNGAYTGGWSNGC | 58.6 | 65.1 | | |
| | | CLINT1_2-3_F2 | 2nd PCR | GGYTTNSAWGGYTGCATNCCNGG | 58.6 | 65.1 | | |
| | | CLINT1_2-3_R2 | | TTSATGGGSACSWSNCARGCNAC | 58.6 | 65.1 | | |
| 15 | CNOT10 | CNOT10_11-12_F1 | 1st PCR | GCRCANGCRTTRTCRTTYTCYTG | 50 | 61 | 1105 | 16(100%) |
| | | CNOT10_11-12_R1 | | GGCAAYTAYMGNAARGCNGTNAA | 45.6 | 59.7 | | |
| | | CNOT10_11-12_F2 | 2nd PCR | TTNCCCATNGCRAARTGDATRCA | 42.7 | 58.5 | | |
| | | CNOT10_11-12_R2 | | GCNGARCAYCCNGGNTTYATGAA | 52.1 | 62 | | |
| 16 | CNOT2 | CNOT2_13-14_F1 | 1st PCR | GCRATAAARCTNGGNMGNTAYGG | 47.8 | 60.6 | 786 | 16(100%) |
| | | CNOT2_13-14_R1 | | CARTCRAARAARTARTANGTNCC | 36.9 | 56.1 | | |
| | | CNOT2_13-14_F2 | 2nd PCR | TAYTAYATGAAYGGNGGNGAYGT | 43.4 | 58.8 | | |
| | | CNOT2_13-14_R2 | | TANGTRTTNGTYTTCATNGTNGG | 39.1 | 57 | | |
| 17 | COPB2 | COPB2_14-15_F1 | 1st PCR | GGNGARATHCARGARATHGTNAA | 39.8 | 57.3 | 777 | 16(100%) |
| | | COPB2_14-15_R1 | | ACYTTRTCNGCCATNSWRAARTC | 43.4 | 58.8 | | |
| | | COPB2_14-15_F2 | 2nd PCR | AAYTAYTAYGTNGGNGGNGARAT | 41.3 | 57.9 | | |
| | | COPB2_14-15_R2 | | ACDATRTTNARYTCYTTRTCNCC | 38.4 | 56.7 | | |
| 18 | CPOX | CPOX_1-2_F1 | 1st PCR | TTYGARAARGCNGGNGTNAAYGT | 45.6 | 59.7 | 1123 | 16(100%) |
| | | CPOX_1-2_R1 | | TCDATYTCRAARTANCKRTARTT | 29.7 | 53.2 | | |
| | | CPOX_1-2_F2 | 2nd PCR | GARGARGCNGCNCARCARATGMG | 58.6 | 65.1 | | |
| | | CPOX_1-2_R2 | | TANGGRTTYTTNGGRTGDATNAC | 40.5 | 57.6 | | |
| 19 | CSTF1 | CSTF1_2-3_F1 | 1st PCR | TCNGTRTGYTGRTCYTGNGGRTT | 50 | 61.5 | 950 | 15(93%) |
| | | CSTF1_2-3_R1 | | GARACNGCNCARCARAAYATGGA | 47.8 | 60.6 | | |
| | | CSTF1_2-3_F2 | 2nd PCR | AARCAYTGRAANGTRTTNACRTC | 36.9 | 56.1 | | |
| | | CSTF1_2-3_R2 | | TAYGAYCAYGTNGAYGARGTNAC | 45.6 | 59.7 | | |
| 20 | CXXC1 | CXXC1_1-2_F1 | 1st PCR | GARAAYGGNGARAAYGCNCCNGT | 54.3 | 63.3 | 1560 | 16(100%) |
| | | CXXC1_1-2_R1 | | GTDATRTTDATRCARTGNCCRTG | 39.8 | 57.3 | | |
| | | CXXC1_1-2_F2 | 2nd PCR | TGYMGNAARCCNGAYATHAAYTG | 42.7 | 58.5 | | |
| | | CXXC1_1-2_R2 | | CCAYTCRTTRCARTGRTCRCANCC | 52 | 63.5 | | |
| 21 | DARS | DARS_8-9_F1 | 1st PCR | GGNCCDATRCARAANACYTTRTC | 44.9 | 59.4 | 1259 | 16(100%) |
| | | DARS_8-9_R1 | | CTTTTYCGNGARACNYTNATHCA | 40.5 | 57.6 | | |
| | | DARS_8-9_F2 | 2nd PCR | AARTCNGCRCADATRCACATYTGYTTRTA | 37.3 | 62.2 | | |
| | | DARS_8-9_R2 | | GGSTTYGTNGARATHCARACNCC | 51.4 | 62.1 | | |
| 22 | DCAF7 | DCAF7_2-3_F1 | 1st PCR | CCNGCNGTRCADATRTGRCA | 54.1 | 59 | 1020 | 15(93%) |
| | | DCAF7_2-3_R1 | | GCNGGNGGNGGNAGNGAYATGTT | 60.8 | 65.9 | | |
| | | DCAF7_2-3_F2 | 2nd PCR | TGNGGNGCCCANGCDATNCCRTT | 60.1 | 65.6 | | |
| | | DCAF7_2-3_R2 | | TAYGARGAYCCNCARCAYCAYCC | 54.3 | 63.3 | | |

| | | | | | | | | |
|---|---|---|---|---|---|---|---|---|
| 23 | DDB1 | DDB1_16-18_F1 | 1st PCR | TGNGCNACRTTNCCRCARAARAA | 45.6 | 59.7 | 848 | 16(100%) |
| | | DDB1_16-18_R1 | | WSHGGNGCNTTYAARGARGG | 54.1 | 59 | | |
| | | DDB1_16-18_F2 | 2nd PCR | TGYTGRTCRTCNACRAANCCNGT | 50 | 61.5 | | |
| | | DDB1_16-18_R2 | | GGNATHGGNATHCAYGARCAYGC | 52.8 | 62.7 | | |
| 24 | DDX18 | DDX18_7-8_F1 | 1st PCR | AAYACNCCNGGNTTYATGTAYAA | 39.1 | 57 | 698 | 16(100%) |
| | | DDX18_7-8_R1 | | CCTTCNACNGTNGCNGTYTCYTT | 52.1 | 62.4 | | |
| | | DDX18_7-8_F2 | 2nd PCR | GARGARATGAARCARATHATHAA | 28.9 | 52.9 | | |
| | | DDX18_7-8_R2 | | TTRTTRTCRTCNACNCCNACRTA | 41.3 | 57.9 | | |
| 25 | DDX23 | DDX23_9-10_F1 | 1st PCR | TTYTAYGGNGAYYTNATGGARAA | 36.9 | 56.1 | 1032 | 15(93%) |
| | | DDX23_9-10_R1 | | TANCCRCAYTTRTCDATNACYTC | 40.5 | 57.6 | | |
| | | DDX23_9-10_F2 | 2nd PCR | YTNGARGARAARGARCARGARGA | 43.47 | 58.8 | | |
| | | DDX23_9-10_R2 | | TCRTCCCANCKYTGYTTNGCYTC | 54.3 | 63.3 | | |
| 26 | DICER1 | DICER1_3-4_F1 | 1st PCR | TCCATRTADATNGCNCCNGC | 51.6 | 57.9 | 821 | 16(100%) |
| | | DICER1_3-4_R1 | | GAYTTYGTNCARTTYCARATGGA | 39.1 | 57 | | |
| | | DICER1_3-4_F2 | 2nd PCR | CCCATNGCYTTNGGNACYTCDAT | 51.4 | 62.1 | | |
| | | DICER1_3-4_R2 | | AARAAYGARATGCARGGNATGGA | 41.3 | 57.9 | | |
| 27 | DLST | DLST_1-2_F1 | 1st PCR | TCYTCNACNACNGCYTTDATYTT | 40.5 | 57.6 | 869 | 15(93%) |
| | | DLST_1-2_R1 | | ATHGARGAYATGGAYGGNGGNAC | 51.4 | 62.1 | | |
| | | DLST_1-2_F2 | 2nd PCR | GCNACRAACATCATNGGNCKNAC | 52.1 | 62.4 | | |
| | | DLST_1-2_R2 | | YTNGGNATGCAYGGNATHTTYGA | 44.9 | 59.4 | | |
| 28 | DNMT1 | DNMT1_16-17_F1 | 1st PCR | TGCATRTCYTCRCAYTCRTCNAC | 47.8 | 60.6 | 1137 | 15(93%) |
| | | DNMT1_16-17_R1 | | TAYTAYCARAARGTNTGYATHGA | 31.8 | 54.1 | | |
| | | DNMT1_16-17_F2 | 2nd PCR | AACCARTGNACRTGRAACATNGG | 45.6 | 59.7 | | |
| | | DNMT1_16-17_R2 | | GARACNYTNCARATTGGNGA | 46.6 | 55.2 | | |
| 29 | DYNC1H1 | DYNC1H1_69-70_F1 | 1st PCR | TTCCANGCRAANARYTCYTGRAA | 41.3 | 57.9 | 1564 | 13(81%) |
| | | DYNC1H1_69-70_R1 | | GGNCARGCNGAYGAYAARGCNGA | 58.6 | 65.1 | | |
| | | DYNC1H1_69-70_F2 | 2nd PCR | GGRTTNARRTADATNACYTGRT | 34 | 55 | | |
| | | DYNC1H1_69-70_R2 | | GAYATGGAYACNGAYGCNCCNCA | 56.5 | 64.2 | | |
| 30 | DYNC2H1 | DYNC2H1_43-44_F1 | 1st PCR | CCCCARTCNCCYTGRAANACYTT | 52 | 62.4 | 1664 | 16(100%) |
| | | DYNC2H1_43-44_R1 | | CGWGCNAARTTYACNGTNGAYGA | 47.8 | 60.6 | | |
| | | DYNC2H1_43-44_F2 | 2nd PCR | CCNACDATYTTRTCNCKRAA | 41.6 | 53.8 | | |
| | | DYNC2H1_43-44_R2 | | CCNTGYATHYTNACNCARTGGGT | 49.2 | 61.2 | | |
| 31 | EIF4G2 | EIF4G2_14-15-F1 | 1st PCR | AAYAARAAYCARGTNCCNAARYT | 34.7 | 55.3 | 734 | 16(100%) |
| | | EIF4G2_14-15-R1 | | TCCTTRSWNGGNGGNGGYTT | 57.5 | 60.3 | | |
| | | EIF4G2_14-15-F2 | 2nd PCR | CARCCNCARATHACNATGATHCC | 46.3 | 60 | | |
| | | EIF4G2_14-15-R2 | | GTYTTNGCNGGYTTYTCYTGDAT | 44.9 | 59.4 | | |
| 32 | ERP44 | ERP44_5-6_F1 | 1st PCR | GGNATGATGATGAARMGNGARTA | 41.3 | 57.9 | 1258 | 12(75%) |
| | | ERP44_5-6_R1 | | GCNARRAANACRCARTCRTCRTG | 47.8 | 60.6 | | |
| | | ERP44_5-6_F2 | 2nd PCR | GCNGAYTAYATHMGNCARCARAA | 42.7 | 58.5 | | |
| | | ERP44_5-6_R2 | | TGYTCRAARTANCCDATDATRTT | 31.1 | 53.8 | | |
| 33 | FOXK2 | FOXK2_5-6_F1 | 1st PCR | GTNCARGCNATHACNATGGCNCC | 55.7 | 63.9 | 1539 | 10(62%) |
| | | FOXK2_5-6_R1 | | CCAGAANGANCCYTTNCCNGGYTC | 58.3 | 66.1 | | |
| | | FOXK2_5-6_F2 | 2nd PCR | ACNCAYATHACNAARAAYTAYCC | 36.2 | 55.8 | | |
| | | FOXK2_5-6_R2 | | GGNACYTTDATRAARTANCKRTT | 34 | 55 | | |
| 34 | FTSJ3 | FTSJ3_2-3_F1 | 1st PCR | AARGGNGGNTGGTTYATHACNAA | 42.7 | 58.5 | 907 | 16(100%) |

| | | | | | | | | |
|---|---|---|---|---|---|---|---|---|
| | | FTSJ3_2-3_R1 | | GCRAAYTTNGGRTCRAARAAYTT | 36.9 | 56 | | |
| | | FTSJ3_2-3_F2 | 2nd PCR | GAYTACCARCCNYTNYTNTGGAT | 45.6 | 59.7 | | |
| | | FTSJ3_2-3_R2 | | TCAATYTTRTCNGGNGCNARRTA | 41.3 | 57.9 | | |
| 35 | GOT1 | GOT1_4-5_F1 | 1st PCR | ACNGAYGCNGGNTTYAARGAYAT | 45.6 | 59.7 | 748 | 15(93%) |
| | | GOT1_4-5_R1 | | TTCCAYTGYTCYTGNGTNGGRTC | 52.1 | 62.4 | | |
| | | GOT1_4-5_F2 | 2nd PCR | GGNYTNGAYATHCARGGNTT | 46.6 | 55.9 | | |
| | | GOT1_4-5_R2 | | GTNGGRTTRTGNGCRCANGCRTG | 58.6 | 65.1 | | |
| 36 | HBS1L | HBS1L_7-8_F1 | 1st PCR | AAYGTNAAYAARMGNACNATGCA | 36.9 | 56.1 | 1193 | 13(81%) |
| | | HBS1L_7-8_R1 | | ATCATRTTNGGDATRAARTCYTT | 29.7 | 53.2 | | |
| | | HBS1L_7-8_F2 | 2nd PCR | AARTAYGARCARGARWSNAARAA | 32.6 | 54.4 | | |
| | | HBS1L_7-8_R2 | | GTCATNCCNACRTCCATNGTNAC | 50 | 61.5 | | |
| 37 | HIPK1 | HIPK1_11-12_F1 | 1st PCR | CAGGCNTGGCCNGGNGGNACNCA | 71.7 | 70.4 | 1438 | 16(100%) |
| | | HIPK1_11-12_R1 | | GTAGCNAGNGTNACRTGRTTNGT | 47.8 | 60.6 | | |
| | | HIPK1_11-12_F2 | 2nd PCR | CAGCCTACNGCNGTNATHCCNGA | 57.9 | 64.8 | | |
| | | HIPK1_11-12_R2 | | TGCATDATNGTGSWRTAYTGRTT | 36.2 | 55.8 | | |
| 38 | HUWE1 | HUWE1_56-57_F1 | 1st PCR | GCNGCCATNGCYGGNGTDATYTT | 55.7 | 63.9 | 962 | 16(100%) |
| | | HUWE1_56-57_R1 | | GARTTYGGNCARGCNGAYATGCA | 52.1 | 62.4 | | |
| | | HUWE1_56-57_F2 | 2nd PCR | GGYTCRTCNGTYTCCATNGG | 57.5 | 60.4 | | |
| | | HUWE1_56-57_R2 | | AAATTNGTNCCNAARGARGARAA | 34.78 | 56.2 | | |
| 39 | INO80 | INO80_9-11_F1 | 1st PCR | CARACNGARYTNTAYGCNCAYTT | 43.4 | 58.8 | 945 | 16(100%) |
| | | INO80_9-11_R1 | | TTCATNCCYTTNARYTGRTANCC | 41.3 | 57.9 | | |
| | | INO80_9-11_F2 | 2nd PCR | AAYATHACNCARGARGAYTAYGG | 40.5 | 57.6 | | |
| | | INO80_9-11_R2 | | SWYTCYTTNGCRTCYTCRTCRAA | 45.6 | 59.7 | | |
| 40 | IPO9 | IPO9_14-15_F1 | 1st PCR | GTNATGGARACNYTNTGYATHGT | 40.5 | 57.6 | 1331 | 11(68%) |
| | | IPO9_14-15_R1 | | CKCATYTGCATNGCRTCYTGRCA | 52.1 | 62.4 | | |
| | | IPO9_14-15_F2 | 2nd PCR | GARAAYAARATHTGYCCNTTYAC | 36.2 | 55.8 | | |
| | | IPO9_14-15_R2 | | ARYTCYTTRAADATRTCYTGNGC | 38.4 | 56.7 | | |
| 41 | JMJD6 | JMJD6_2-3_F1 | 1st PCR | GCNCARGARAARTGGACNYTNGA | 50 | 61.5 | 1679 | 15(93%) |
| | | JMJD6_2-3_R1 | | TCNCCNGGYTTYTGNACDATYTC | 49.2 | 61.2 | | |
| | | JMJD6_2-3_F2 | 2nd PCR | AAYCARAARTTYAARTGYGGNGA | 36.9 | 56.1 | | |
| | | JMJD6_2-3_R2 | | AACCANGTDATNGCYTCRTCYTG | 47.1 | 60.3 | | |
| 42 | KIF3A | KIF3A_12-13_F1 | 1st PCR | AAAGTGATHGTNGGNGGNGTNGA | 49.2 | 61.2 | 1546 | 13(81%) |
| | | KIF3A_12-13_R1 | | GCCATNARCATNGTCCANACYTT | 45.6 | 59.7 | | |
| | | KIF3A_12-13_F2 | 2nd PCR | GCNAARGCNGARGARCARGARAA | 50 | 61.5 | | |
| | | KIF3A_12-13_R2 | | GTYTTNCCYTGNGCYTCYTCYTG | 54.3 | 63.3 | | |
| 43 | KLHL24 | KLHL24_2-3_F1 | 1st PCR | CCNTAYACNGARTGYTAYGAYCC | 50 | 61.5 | 833 | 13(81%) |
| | | KLHL24_2-3_R1 | | TGNCKCCANCKNCCYTTRTT | 52.5 | 58.3 | | |
| | | KLHL24_2-3_F2 | 2nd PCR | GARTTYACNAARWSNGARTAYGC | 41.3 | 57.9 | | |
| | | KLHL24_2-3_R2 | | TGNSWRTTRTADATCCANACRTC | 38.4 | 56.7 | | |
| 44 | LYST | LYST_45-46-F1 | 1st PCR | YTNAARTGGGGNGARTAYGTNGG | 50 | 61.5 | 1722 | 13(81%) |
| | | LYST_45-46-F2 | | CARCCRCAYGGNGARMGNTTYGG | 60.8 | 65.9 | | |
| | | LYST_45-46-R1 | 2nd PCR | ARDATRTTRTCNGCRTANCCCCA | 44.9 | 59.4 | | |
| | | LYST_45-46-R2 | | CCAYTGDATRTCNGTNSWRTGCAT | 45.1 | 60.7 | | |
| 45 | LZTR1 | LZTR1_14-15_F1 | 1st PCR | TNYTNATHATGGAYGTNTAYAA | 28.7 | 51.5 | 911 | 14(87%) |
| | | LZTR1_14-15_R1 | | CATCATDATNACYTGRTTRAARTG | 32.6 | 55.5 | | |

| | | | | | | | | |
|---|---|---|---|---|---|---|---|---|
| | | LZTR1_14-15_F2 | 2nd PCR | GTNGTNTGYGARAAYGCNAAYAA | 41.3 | 57.9 | | |
| | | LZTR1_14-15_R2 | | ACNACRAARTTNARRCARTGYTC | 39.1 | 57 | | |
| 46 | MCM2 | MCM2_3-4_F1 | 1st PCR | AAYTTYTGNGTRTCDATRAA | 29.1 | 48.7 | 1265 | 15(93%) |
| | | MCM2_3-4_R1 | | AARAARTAYATHATHTAYGCNAA | 22.4 | 50.2 | | |
| | | MCM2_3-4_F2 | 2nd PCR | ATNGCCATRTTNACRTCRTCYTC | 43.4 | 58.8 | | |
| | | MCM2_3-4_R2 | | CARATGGAYCARGAYAARGTNGC | 47.8 | 60.6 | | |
| 47 | MFSD4 | MFSD4_6-7_F1 | 1st PCR | TTYGGRTGYTGYCARWSNAARAA | 41.3 | 57.9 | 1608 | 16(100%) |
| | | MFSD4_6-7_R1 | | ACRTTDATRAANACCATNGTNGC | 38.4 | 56.7 | | |
| | | MFSD4_6-7_F2 | 2nd PCR | YTNTTYATGACNGAYGGNAT | 40 | 53.2 | | |
| | | MFSD4_6-7_R2 | | CCNARNGTDATRAANCCCCARAA | 44.9 | 59.4 | | |
| 48 | MPDZ | MPDZ_4-5_F1 | 1st PCR | TCAATNGTSCCNARNGCRTTYTT | 43.4 | 58.8 | 1529 | 12(75%) |
| | | MPDZ_4-5_R1 | | GGNGAYGTWCCSATHTTYATHGC | 48.5 | 60.9 | | |
| | | MPDZ_4-5_F2 | 2nd PCR | ACWGCCTGNSWRTGNGTCATNCC | 56.5 | 64.2 | | |
| | | MPDZ_4-5_R2 | | ATGCAYCCNAAYGGNGTNGCNGC | 60.8 | 65.9 | | |
| 49 | MYO1D | MYO1D_7-8_F1 | 1st PCR | TCYTGRAANCCRCARTGYTCDAT | 44 | 59.4 | 1090 | 15(93%) |
| | | MYO1D_7-8_R1 | | CCCAAYGAYAARAARWSNCCNCA | 47 | 60.6 | | |
| | | MYO1D_7-8_F2 | 2nd PCR | TTRTGRTTNGGCCANGTRAAYTC | 43.4 | 58.8 | | |
| | | MYO1D_7-8_R2 | | CAYCARGTNGARTAYYTNGG | 47.5 | 56.2 | | |
| 50 | MYOF | MYOF_36-37_F1 | 1st PCR | TGGGGNYTNMGNAAYATGAARAA | 41.3 | 57.9 | 1187 | 15(93%) |
| | | MYOF_36-37_R1 | | TCYTTNGTNGCRTANGGRTCRCA | 50 | 61.5 | | |
| | | MYOF_36-37_F2 | 2nd PCR | AARAAYTAYCARATGGCNWSNGT | 39.1 | 57 | | |
| | | MYOF_36-37_R2 | | AAYTGNCKRTGRTCDATNACYTT | 38.4 | 56.7 | | |
| 51 | NIPBL | NIPBL_6-7_F1 | 1st PCR | TTDATNGCYTGYTGNACYTGRTA | 40.5 | 57.6 | 830 | 16(100%) |
| | | NIPBL_6-7_R1 | | ATHGCNATGGGNACNGAYCCNGA | 55.7 | 63.9 | | |
| | | NIPBL_6-7_F2 | 2nd PCR | TTCATNCCNGCNACNGCYTTCAT | 50 | 61.5 | | |
| | | NIPBL_6-7_R2 | | AARAARTAYGCNGGNTTYATHCA | 36.2 | 55.8 | | |
| 52 | NUTM1 | NUTM1_1-2_F1 | 1st PCR | AARGGNGTNTAYGARAAYTAYMG | 39.1 | 57 | 1699 | 16(100%) |
| | | NUTM1_1-2_R1 | | TTYTCNGCCATYTCRTARAADAT | 34 | 55 | | |
| | | NUTM1_1-2_F2 | 2nd PCR | TGGCARMGYTAYAARGCNYTNGC | 52.1 | 62.4 | | |
| | | NUTM1_1-2_R2 | | TGYTCCCAYTCYTGNACNGCNCK | 58.6 | 65.1 | | |
| 53 | PDXK | PDXK_3-4_F1 | 1st PCR | TTRTCNGGRTGYTTRTGNGTCCA | 47.8 | 60.6 | 1433 | 11(68%) |
| | | PDXK_3-4_R1 | | ATGGAYATGTYNCAYAAYATGGG | 41.3 | 57.9 | | |
| | | PDXK_3-4_F2 | 2nd PCR | CCNACRAANACNGCRTCNACYTT | 50 | 61.5 | | |
| | | PDXK_3-4_R2 | | CCNGARACNGTNGTNATHAC | 49.1 | 56.9 | | |
| 12 | POLR1B | POLR1B_4-5 | 1st PCR | CCCATYTGRCAYTGRTACATRTT | 41.3 | 57.9 | 1609 | 12(75%) |
| | | POLR1B_4-5 | | TAYCCRGGNYTNTTYATHTTYAC | 38.4 | 56.7 | | |
| | | POLR1B_4-5 | 2nd PCR | TGRTTRTGRTCNSWRAANGG | 45 | 55.2 | | |
| | | POLR1B_4-5 | | GARTGGATHGGNACNATGGARCA | 49.2 | 61.2 | | |
| 54 | POLR2B | POLR2B_5-6_F1 | 1st PCR | TAYGTNGAYATHACNAARACNGT | 36.2 | 55.8 | 835 | 15(93%) |
| | | POLR2B_5-6_R1 | | TCYTTYTTNGCRAANACRTANAC | 36.9 | 56.1 | | |
| | | POLR2B_5-6_F2 | 2nd PCR | CARACNCARCAYCARAARACNTT | 41.3 | 57.9 | | |
| | | POLR2B_5-6_R2 | | GTNGCCATYTTYTCYTGNGCDAT | 47.1 | 60.3 | | |
| 55 | PRPF38A | PRPF38A_3-5_F1 | 1st PCR | GGNACNGCNATHGAYTGYTAYAA | 44.9 | 59.4 | 923 | 15(93%) |
| | | PRPF38A_3-5_R1 | | TCNACRTCRTCCATRTCYTCYTC | 47.8 | 60.6 | | |
| | | PRPF38A_3-5_F2 | 2nd PCR | TAYAAYGAYTAYMGNAARATHAA | 25.3 | 51.4 | | |

| | | | | | | | | |
|---|---|---|---|---|---|---|---|---|
| | | PRPF38A_3-5_R2 | | TGGTCNGCYTCYTCNARNACRTA | 50 | 61.5 | | |
| 56 | PRR7 | PRR7_1-2_F1 | 1st PCR | ARRTGCCANGGNCCNGGYTCRTA | 58.6 | 65.1 | 1184 | 15(93%) |
| | | PRR7_1-2_R1 | | ACNTGYTTYGCNGGNTTYTGGYT | 50 | 61.5 | | |
| | | PRR7_1-2_F2 | 2nd PCR | GGYTGYTCYTGYTTYTCNGGYTG | 54.3 | 63.3 | | |
| | | PRR7_1-2_R2 | | CAYTAYGARGGNTAYGGNGGNCC | 58.6 | 65.1 | | |
| 57 | PSMD12 | PSMD12_3-4_F1 | 1st PCR | TCYTTCCANCKYTTYTCNCCYTC | 50 | 61.5 | 634 | 12(75%) |
| | | PSMD12_3-4_R1 | | CCYTAYGAYAAYGARCARWSNGA | 45.6 | 59.7 | | |
| | | PSMD12_3-4_F2 | 2nd PCR | CKCATNARYTCCATNGTNGTRAA | 41.3 | 57.9 | | |
| | | PSMD12_3-4_R2 | | YTNGARGAYATHCCNAARTAYAA | 34 | 55 | | |
| 58 | PTPRQ | PTPRQ_4-5_F1 | 1st PCR | TCNGGNACNCCRTGYTCNGGCCA | 65.2 | 67.7 | 1167 | 10(62%) |
| | | PTPRQ_4-5_R1 | | TGYCAYCARTAYTGGCCNGARGA | 52.1 | 62.4 | | |
| | | PTPRQ_4-5_F2 | 2nd PCR | GTRAARTTRCAYTGNCKNACCAT | 41.3 | 57.9 | | |
| | | PTPRQ_4-5_R2 | | CARATHGAYTGGACNATHMGNGA | 44.2 | 59.1 | | |
| 59 | PXK | PXK_9-10_F1 | 1st PCR | TTDATYTTNCKRAAYTGNGTRAA | 29.7 | 53.2 | 790 | 11(68%) |
| | | PXK_9-10_R1 | | AARAARTAYTGYAAYCCNAARAA | 28.2 | 52.6 | | |
| | | PXK_9-10_F2 | 2nd PCR | AANCCYTTYTCRTGNARRAAYTT | 34.7 | 55.3 | | |
| | | PXK_9-10_R2 | | YTNCARCARATHAARACNTTYGG | 38.4 | 56.7 | | |
| 60 | RALGAPB | RALGAPB_6-7_F1 | 1st PCR | TTYAARATHCCNGAYGARGAYGC | 44.9 | 59.4 | 1081 | 16(100%) |
| | | RALGAPB_6-7_R1 | | CKCATNGCNCKRAARAADATYTG | 42.7 | 58.5 | | |
| | | RALGAPB_6-7_F2 | 2nd PCR | CCNCCNGARATGGAYAAYGARTG | 52.1 | 62.4 | | |
| | | RALGAPB_6-7_R2 | | AAYTGYTCYTGRAAYTTNGGNGT | 41.3 | 57.9 | | |
| 61 | RBM22 | RBM22_5-6_F1 | 1st PCR | CAYGARAARCCNACNGAYCCNGA | 54.3 | 63.3 | 970 | 15(93%) |
| | | RBM22_5-6_R1 | | GCRAAYTGDATRAANGCRCAYTG | 44.9 | 59.4 | | |
| | | RBM22_5-6_F2 | 2nd PCR | AAYATHAARGAYMGNTAYTAYGG | 34 | 55 | | |
| | | RBM22_5-6_R2 | | TCNCCRAAYTGRTARAARTGRTT | 36.9 | 56.1 | | |
| 62 | RIOK3 | RIOK3_8-9_F1 | 1st PCR | GTNSWRTGYTCYTTYTTYTCRTG | 41.3 | 57.9 | 1079 | 16(100%) |
| | | RIOK3_8-9_R1 | | CCTACNACNACNCCNAARAARGG | 52.1 | 62.4 | | |
| | | RIOK3_8-9_F2 | 2nd PCR | TCCATNCCDATNCCRTCNCCNAC | 55.7 | 63.9 | | |
| | | RIOK3_8-9_R2 | | GGNAARGGNAARGAYATHACNAC | 44.9 | 59.4 | | |
| 63 | RUVBL2 | RUVBL2_7-9_F1 | 1st PCR | TAYGAYYTNGGNACNAARATGAT | 36.9 | 56.1 | 1181 | 16(100%) |
| | | RUVBL2_7-9_R1 | | TTDATNACRTCDATYTCRTG | 33.3 | 50.4 | | |
| | | RUVBL2_7-9_F2 | 2nd PCR | ACNAARGARAARGTNCARGCNGG | 50 | 61.5 | | |
| | | RUVBL2_7-9_R2 | | TCNGGRCAYTGNACRAAYTTNGT | 45.6 | 59.7 | | |
| 64 | SAP18 | SAP18_2-3_F1 | 1st PCR | GTNAARGARGTNTAYCCNGARGC | 50 | 61.5 | 1271 | 16(100%) |
| | | SAP18_2-3_R1 | | TTYGGWGGNGTDATNGCDATRTC | 46.3 | 60 | | |
| | | SAP18_2-3_F2 | 2nd PCR | GGNACNCAYTTYAAYTTYGCNAT | 41.3 | 57.9 | | |
| | | SAP18_2-3_R2 | | TARTCNCCDATYTGRAAYTTYTG | 36.2 | 55.8 | | |
| 65 | SEC13 | SEC13_6-7_F1 | 1st PCR | GGYTGCAAYGCNGTNWSNTGGGC | 63 | 66.8 | 1005 | 14(87%) |
| | | SEC13_6-7_R1 | | GCCCANGCNACRTCNCKNACCCA | 65.2 | 67.7 | | |
| | | SEC13_6-7_F2 | 2nd PCR | CARAARCCNAAYTAYATHAARMG | 34 | 55 | | |
| | | SEC13_6-7_R2 | | TCYTTCCAYTGNCCRTCYTCYTC | 52.1 | 62.4 | | |
| 66 | SF3B1 | SF3B1_18-19_F1 | 1st PCR | GGNGCNGCNGAYATHGAYCAYAA | 53.6 | 63 | 724 | 14(87%) |
| | | SF3B1_18-19_R1 | | TCYTGRCANGTYTTCATNACNAC | 43.4 | 58.8 | | |
| | | SF3B1_18-19_F2 | 2nd PCR | ATHYTNTAYGCNTTYCARGARCA | 38.4 | 56.7 | | |
| | | SF3B1_18-19_R2 | | GTRCCRAANCCRTTNARCATNAC | 45.6 | 59.7 | | |

| | | | | | | | | |
|---|---|---|---|---|---|---|---|---|
| 67 | SKIV2L2 | SKIV2L2_22-23_F1 | 1st PCR | TTDATNGTNACRTCNCCNGTCAT | 42.7 | 58.5 | 1171 | 16(100%) |
| | | SKIV2L2_22-23_R1 | | GARTAYCCNTTYATHYTNGAYGC | 42.7 | 58.5 | | |
| | | SKIV2L2_22-23_F2 | 2nd PCR | TCYTGRAAYTCYTCRTACATYTC | 39.1 | 57 | | |
| | | SKIV2L2_22-23_R2 | | GCWATHYTNTGYGTNGAYAAYAA | 36.2 | 55.8 | | |
| 68 | SLU7 | SLU7_14-15_F1 | 1st PCR | ACNCCNCKYTTRTACCAYTCNCC | 54.3 | 63.3 | 1113 | 15(93%) |
| | | SLU7_14-15_R1 | | GARGAYTGGMGNAARAARAARGA | 41.3 | 57.9 | | |
| | | SLU7_14-15_F2 | 2nd PCR | TGNGGDATRTGNGGRTTDATRTC | 44.2 | 59.1 | | |
| | | SLU7_14-15_R2 | | GCNGARGTNGAYGARGARGGNAA | 54.3 | 63.3 | | |
| 69 | SMC2 | SMC2_22-23_F1 | 1st PCR | AARMGNATHGTNGARAAYGAYAA | 34 | 55 | 1464 | 15(93%) |
| | | SMC2_22-23_R1 | | ARRTTYTCYTTCCANGTRTTNCC | 41.3 | 57.9 | | |
| | | SMC2_22-23_F2 | 2nd PCR | YTNGAYCARAARAARAAYGARGC | 39.1 | 57 | | |
| | | SMC2_22-23_R2 | | GCRACYTTRAAYTCNARNCCRTC | 47.8 | 60.6 | | |
| 70 | SRP72 | SRP72_3-5_F1 | 1st PCR | GTCATNGCNCCYTGNGTNCCYTT | 56.5 | 64.2 | 1335 | 16(100%) |
| | | SRP72_3-5_R1 | | AARAARGGNGGNAARCCNAC | 50 | 57.3 | | |
| | | SRP72_3-5_F2 | 2nd PCR | CCDATYTGRTCYTTYTTYTTNCC | 40.57 | 57.6 | | |
| | | SRP72_3-5_R2 | | GARAAYCARCARAARGARCARGG | 45.6 | 59.7 | | |
| 71 | SSRP1 | SSRP1_5-6_F1 | 1st PCR | AARGCNGAYGTNATHCARGCNAC | 49.2 | 61.2 | 676 | 15(93%) |
| | | SSRP1_5-6_R1 | | ATRTCYTCRTCYTTNSWRAA | 35 | 51.1 | | |
| | | SSRP1_5-6_F2 | 2nd PCR | CAYAARGAYCARMGNCARATGTT | 36.9 | 56.1 | | |
| | | SSRP1_5-6_R2 | | TGNCCYTGYTTDATNGGNGGRTC | 53.6 | 63 | | |
| 72 | SULF1 | SULF1_7-8_F1 | 1st PCR | GGNTAYCAYATHGGNCARTTYGG | 49.2 | 61.2 | 697 | 16(100%) |
| | | SULF1_7-8_R1 | | TTNCCRTCCATRTCNGGNGGNGT | 56.5 | 64.2 | | |
| | | SULF1_7-8_F2 | 2nd PCR | WSNATGCCNTAYGAYTTYGAYAT | 39.1 | 57 | | |
| | | SULF1_7-8_R2 | | ACYGTYGGNGCNARRTCDATRTT | 47.1 | 60.3 | | |
| 73 | TAF2 | TAF2_10-11_F1 | 1st PCR | TTYTTNCKRTTRTCRTTRTAYTT | 26 | 51.7 | 1026 | 13(81%) |
| | | TAF2_10-11_R1 | | AAYTTYATGGTNWSNACNTGGAC | 41.3 | 57.9 | | |
| | | TAF2_10-11_F2 | 2nd PCR | ACYTCYTTNGGRCANARRTTRTG | 43.4 | 58.8 | | |
| | | TAF2_10-11_R2 | | TTYACNMGNATGTTYTGYTGYAA | 36.9 | 56.1 | | |
| 74 | TBC1D16 | TBC1D16_9-10_F1 | 1st PCR | CARTTYACNGTNGAYAARGAYGT | 41.3 | 57.9 | 871 | 16(100%) |
| | | TBC1D16_9-10_R1 | | SWDATRAADATNGTRTTYTGCAT | 28.9 | 52.9 | | |
| | | TBC1D16_9-10_F2 | 2nd PCR | GGNGARAAYAAYCCNAAYGTNGA | 45.6 | 59.7 | | |
| | | TBC1D16_9-10_R2 | | CACCARAANGTRTCNSWYTCRTC | 47.8 | 60.6 | | |
| 75 | TENM3 | TENM3_5-6_F1 | 1st PCR | ACYTTNCCYTGNGCYTGYTCYTT | 50 | 61.5 | 763 | 15(93%) |
| | | TENM3_5-6_R1 | | AAYGTNACNTTYCCNACNGG | 50 | 57.3 | | |
| | | TENM3_5-6_F2 | 2nd PCR | TGNGGYTCNGTYTGRTARTGNGT | 50 | 61.5 | | |
| | | TENM3_5-6_R2 | | ATHGAYWSNTTYTAYACNATGGT | 34 | 55 | | |
| 76 | TMCO3 | TMCO3_2-3_F1 | 1st PCR | GCNGARAARCAYCARGCNGARGC | 58.6 | 65.1 | 900 | 15(93%) |
| | | TMCO3_2-3_R1 | | TTDATRAARTGRTGRTCNGCNCK | 42.7 | 58.5 | | |
| | | TMCO3_2-3_F2 | 2nd PCR | GARCAYGCNTTYGAYGAYAAYAA | 41.3 | 57.9 | | |
| | | TMCO3_2-3_R2 | | TAYTGRTTRTTYTGNSWRTCDAT | 31.8 | 54.1 | | |
| 77 | TOMM70A | TOMM70A_4-5_F1 | 1st PCR | GARGARAAYTAYGAYAARATHAT | 27.5 | 52.3 | 1240 | 15(93%) |
| | | TOMM70A_4-5_R1 | | ACRTCNGCRTTYTGNGGRTCDAT | 49.2 | 61.2 | | |
| | | TOMM70A_4-5_F2 | 2nd PCR | GARTGYACNAARGARATHGARGC | 44.9 | 59.4 | | |
| | | TOMM70A_4-5_R2 | | GCNGCCATRTTRAARTCYTGNGT | 47.8 | 60.6 | | |
| 78 | TRAPPC11 | TRAPPC11_28-29_F1 | 1st PCR | TGYTTYTCYTTCCAYTGNGGYTC | 47.8 | 60.6 | 754 | 15(93%) |

| | | | | | | | | |
|---|---|---|---|---|---|---|---|---|
| | | TRAPPC11_28-29_R1 | | CARTGGGAYTTYCCNGTNGARYT | 50 | 61.5 | | |
| | | TRAPPC11_28-29_F2 | 2nd PCR | CCYTTNGGDATRTACCAYTCRTA | 42.7 | 58.5 | | |
| | | TRAPPC11_28-29_R2 | | GTNTGGGAYGCNTTYTGYGCNAA | 52.1 | 62.4 | | |
| 79 | TTN | TTN_89-90_F1 | 1st PCR | CTCAARGAYGTNACNGCNAARGA | 47.8 | 60.6 | 1606 | 15(93%) |
| | | TTN_89-90_R1 | | GCNGANGTYTGRTCNGTNCC | 60 | 61.4 | | |
| | | TTN_89-90_F2 | 2nd PCR | ATGAARGAYGAYGGNAARTTYCA | 39.1 | 57 | | |
| | | TTN_89-90_R2 | | TCYTGNCCRTCYTTRAACCAYTT | 43.4 | 58.8 | | |
| 80 | U2AF2 | U2AF2_11-12_F1 | 1st PCR | CARGTNCARATGGGNGGNCAYCC | 60.8 | 65.9 | 1133 | 16(100%) |
| | | U2AF2_11-12_R1 | | TCNGGRTCRCARTAYTTNGTNAC | 45.6 | 59.7 | | |
| | | U2AF2_11-12_F2 | 2nd PCR | TAYGARGARATHGTNGARGAYGT | 40.5 | 57.6 | | |
| | | U2AF2_11-12_R2 | | TGCATNGCYTTYTGRCARTCRAA | 43.4 | 58.8 | | |
| 81 | UBA1 | UBA1_19-22_F1 | 1st PCR | ACYTTDATYTCCATNGGYTCRCA | 42.7 | 58.5 | 1280 | 14(87%) |
| | | UBA1_19-22_R1 | | TAYYTNCGWGAGGARGAYYTNGG | 50 | 61.5 | | |
| | | UBA1_19-22_F2 | 2nd PCR | TCRTCNAGRCASGTNACYTCNCC | 56.5 | 64.2 | | |
| | | UBA1_19-22_R2 | | CCWGTWACWGCNTAYACNGGNCC | 56.5 | 64.2 | | |
| 82 | UBE3A | UBE3A_4-5_F1 | 1st PCR | TCRTTNGTNACCATRTGRAANCC | 43.4 | 58.8 | 1467 | 16(100%) |
| | | UBE3A_4-5_R1 | | CARTAYGARGGNASNGTRGARGA | 50 | 61.5 | | |
| | | UBE3A_4-5_F2 | 2nd PCR | AANGCYTTRAAYTGYTTYTCNAC | 36.9 | 56.1 | | |
| | | UBE3A_4-5_R2 | | ACNTTCCARATHTSNCAYACNGA | 42.7 | 58.5 | | |
| 83 | UBR5 | UBR5_13-14_F1 | 1st PCR | GAYGGNAAYGGNACNATHTAYCC | 49.2 | 61.2 | 898 | 16(100%) |
| | | UBR5_13-14_R1 | | TCYTCYTCYTTNGTYTCYTTRTT | 36.9 | 56.1 | | |
| | | UBR5_13-14_F2 | 2nd PCR | ATGGCNAARGAYTGYATGGGNGG | 54.34 | 63.3 | | |
| | | UBR5_13-14_R2 | | ATRTGYTGCATNARNGTYTGYTT | 36.9 | 56.1 | | |
| 84 | UBXN7 | UBXN7_4-6_F1 | 1st PCR | TGNCCRTCNARYTGNCCRTGYTC | 56.5 | 64.2 | 1378 | 16(100%) |
| | | UBXN7_4-6_R1 | | GCNAARGARTGYGGNCARATGCA | 52.1 | 62.4 | | |
| | | UBXN7_4-6_F2 | 2nd PCR | ACRTCNARYTGRTGCCAYTCNAC | 50 | 61.5 | | |
| | | UBXN7_4-6_R2 | | GTNCARGAYTTYGCNTGYCARTG | 50 | 61.5 | | |
| 85 | UGCG | UGCG_4-5_F1 | 1st PCR | GGNAARAARGTNGGNATHAAYCC | 44.9 | 59.4 | 1547 | 15(93%) |
| | | UGCG_4-5_R1 | | ACYTTYTCNGTCATYTGRTTNGC | 43.4 | 58.8 | | |
| | | UGCG_4-5_F2 | 2nd PCR | GCNTAYGARGTNGCNAARTAYGA | 45.6 | 59.7 | | |
| | | UGCG_4-5_R2 | | GCCATRTCYGTNARNGTRTCNGG | 54.3 | 63.3 | | |
| 86 | USP14 | USP14_2-4_F1 | 1st PCR | AARTGGGGNAARGARAARTTYGA | 39.1 | 57 | 1592 | 15(93%) |
| | | USP14_2-4_R1 | | GCNARYTGYTCYTCNGTCATRTC | 50 | 61.5 | | |
| | | USP14_2-4_F2 | 2nd PCR | CCNCCNATGGTNTTYAARGCNCA | 52.1 | 62.4 | | |
| | | USP14_2-4_R2 | | GGHCKNGCNACNGGYTCYTC | 66.6 | 64.1 | | |
| 87 | USP34 | USP34_75-77_F1 | 1st PCR | GGNAARGAYGARGTNTAYGAYMG | 47.8 | 60.6 | 1662 | 14(87%) |
| | | USP34_75-77_R1 | | CKYTCRTCCATNARDATRCAYTT | 38.4 | 56.7 | | |
| | | USP34_75-77_F2 | 2nd PCR | GTNGCNATHAAYTGYGARAARTT | 36.2 | 55.8 | | |
| | | USP34_75-77_R2 | | TCNGCRAANACNGGRTCYTCRCA | 54.3 | 63.3 | | |
| 88 | VPS13B | VPS13B_36-37_F1 | 1st PCR | SWYTCDATYTTRCAYTTNACYTT | 31.8 | 54.1 | 1337 | 13(81%) |
| | | VPS13B_36-37_R1 | | GARCARACAACNWSNAAYATHGG | 42.7 | 58.5 | | |
| | | VPS13B_36-37_F2 | 2nd PCR | GTRTANACRTCYTGNACRTCNAC | 45.6 | 59.7 | | |
| | | VPS13B_36-37_R2 | | GCNCCNGAYCCNGARAAYAARGG | 58.6 | 65.1 | | |
| 89 | VPS54 | VPS54_6-7_F1 | 1st PCR | CCNTGGTSNCAYTTYAAYACNGC | 52 | 62.4 | 831 | 15(93%) |
| | | VPS54_6-7_R1 | | TGYTGNGWNGTCATNGCRTGRAA | 47.8 | 60.6 | | |

| | | | | | | | | |
|---|---|---|---|---|---|---|---|---|
| | | VPS54_6-7_F2 | 2nd PCR | AARGGNAAYMGNGAYGCNGC | 57.5 | 60.3 | | |
| | | VPS54_6-7_R2 | | ATYTGRTGNGCDATRTTNACYTC | 40.5 | 57.6 | | |
| 90 | WDFY3 | WDFY3_62-63_F1 | 1st PCR | TCAGGNACRTGNGGNARRTCRAA | 50 | 61.5 | 1491 | 14(87%) |
| | | WDFY3_62-63_R1 | | GCCATHGTNCARTTYYTNGARAT | 40.5 | 57.6 | | |
| | | WDFY3_62-63_F2 | 2nd PCR | GTNGTCATRCARTCNACNGTYTT | 43.4 | 58.8 | | |
| | | WDFY3_62-63_R2 | | GGTTGGATGYTNYTNACNACNAT | 43.4 | 58.8 | | |
| 91 | WDR36 | WDR36_7-8_F1 | 1st PCR | GTNCAYAAYATHAARTTYGAYGA | 31.8 | 54.1 | 686 | 14(87%) |
| | | WDR36_7-8_R1 | | TCNCCYTGNACRAANSACATRCC | 52.1 | 62.4 | | |
| | | WDR36_7-8_F2 | 2nd PCR | AARTTYCARCARGAYTGGGGNCC | 52.1 | 62.4 | | |
| | | WDR36_7-8_R2 | | TCCCANARNCCDATRTGNCCAAC | 51.4 | 62.1 | | |
| 92 | WDR47 | WDR47_13-14_F1 | 1st PCR | ACNTGGWSNGGNTGGATGATHGC | 55.7 | 63.9 | 1340 | 16(100%) |
| | | WDR47_13-14_R1 | | TCNGTNACYTTDATYTTCATRTC | 34 | 55 | | |
| | | WDR47_13-14_F2 | 2nd PCR | GAYAARACNGTNMGNTTYTGGGA | 45.6 | 59.7 | | |
| | | WDR47_13-14_R2 | | TGNGGRTGRTANSWYTGNACCAT | 47.82 | 60.6 | | |
| 93 | YIPF3 | YIPF3_2-3_F1 | 1st PCR | GCNGAYGARGARGAYGGNGARTT | 54.3 | 63.3 | 1552 | 10(62%) |
| | | YIPF3_2-3_R1 | | TGRTTNGGYTCNACRTCRAARTA | 41.3 | 57.9 | | |
| | | YIPF3_2-3_F2 | 2nd PCR | GGNATGAARGGNTTYAARGGNCA | 47.8 | 60.6 | | |
| | | YIPF3_2-3_R2 | | TCDATRTTNGCRTANARRTTRAA | 29.7 | 53.2 | | |
| 94 | ZNF142 | ZNF142_4-5_F1 | 1st PCR | TTYAARACNCAYATGTGYCCNGA | 43.4 | 58.8 | 2174 | 15(93%) |
| | | ZNF142_4-5_R1 | | TARTGYTTCCAYTTNGTNACRTA | 34.7 | 55.3 | | |
| | | ZNF142_4-5_F2 | 2nd PCR | CARTGYCCNAAYTGYCARAARTT | 41.3 | 57.9 | | |
| | | ZNF142_4-5_R2 | | ARYTGRCAYTTRTGNGGYTTYTC | 43.4 | 58.8 | | |
| 95 | ZNF608 | ZNF608_3-4_F1 | 1st PCR | TGRAANGGRTCRTAYTGNCCNGG | 54.3 | 63.3 | 1663 | 16(100%) |
| | | ZNF608_3-4_R1 | | GGNTTYCAYTAYCCNGTNTAYGG | 50 | 61.5 | | |
| | | ZNF608_3-4_F2 | 2nd PCR | TANCCCATNCCNACRTGNGTRTG | 52.1 | 62.4 | | |
| | | ZNF608_3-4_R2 | | TNCARCARCAYGCNAAYCARTA | 43.1 | 57.4 | | |
| 96 | ZSWIM8 | ZSWIM8_26-27_F1 | 1st PCR | GCNCAYAAYGAYCAYCCNAAYAA | 45.6 | 59.7 | 1326 | 15(93%) |
| | | ZSWIM8_26-27_R1 | | TGRTTRTGNACRTGNGCNGGRTT | 50 | 61.5 | | |
| | | ZSWIM8_26-27_F2 | 2nd PCR | TAYACNGARGAYGTNAARTGGYT | 41.3 | 57.9 | | |
| | | ZSWIM8_26-27_R2 | | TTNGCNGCNCCNACRCARAAYTG | 54.3 | 63.3 | | |

*Product size is according to the location of the second-round primers mapping to the king cobra's sequences.
